# Supplementary material for: The impact of continuous quality improvement on coverage of antenatal HIV care tests in rural South Africa: Results of a stepped-wedge cluster-randomised controlled implementation trial
Source: PLoS Med. 2020 Oct 7;17(10):e1003150. doi: 10.1371/journal.pmed.1003150 (PMC7540892; doi:10.1371/journal.pmed.1003150)
Supplement: S1 CONSORT Checklist — CONSORT, Consolidated Standards of Reporting Trials. (DOCX) [file pmed.1003150.s001.docx]

**Supplementary File 2**

**“The impact of Continuous Quality Improvement on coverage of antenatal HIV care tests in rural South Africa: results of a stepped-wedge cluster-randomised controlled implementation trial”**

**CONSORT CHECKLIST FOR STEPPED WEDGE TRIALS**

| **Topic** | **Item number** | **Checklist item** | **Section** |
| --- | --- | --- | --- |
| **Title and abstract** | | | |
|  | 1a | Identification as a SW-CRT in title | Section 1 |
|  | 1b | Structured summary of trial design, methods, results, and conclusions | Section 1 |
| **Introduction** | | | |
| Background and objectives | 2a | Scientific background. Rationale for using a cluster design and rationale for using a stepped wedge design | Section 2 (Introduction) paragraphs 1-5;  study protocol paper |
|  | 2b | Specific objectives or hypotheses | Section 2 (Introduction) paragraph 6; study protocol paper |
| **Methods** | | | |
| Trial design | 3a | Description and diagram of trial design including definition of cluster, number of sequences, number of clusters randomized to each sequence, number of periods, duration of time between each step, and whether the participants assessed in different periods are the same people, different people or a mixture | Section 3 (Methods), paragraph 3; Figure 2; study protocol paper |
|  | 3b | Important changes to methods after trial commencement (such as eligibility criteria), with reasons | Not applicable |
| Participants | 4a | Eligibility criteria for clusters and participants | Section 3 (Methods), paragraph 8-9 |
|  | 4b | Settings and locations where the data were collected | Section 3 (Methods), paragraph 2; study protocol paper, S1 Text, S1 Table, S2 Table |
| Interventions | 5 | The intervention and control conditions with sufficient details to allow replication, including whether the intervention was maintained or repeated, and whether it was delivered at the cluster level, the individual participant level, or both. | Section 3 (Methods), paragraphs 5-8; S1 Text; study protocol paper |
| Outcomes | 6a | Completely defined prespecified primary and secondary outcome measures, including how and when they were assessed. | Section 3 (Methods), paragraphs 11-12; paragraphs 16-17; study protocol paper |
|  | 6b | Any changes to trial outcomes after the trial commenced, with reasons. | Not applicable |
| Sample size | 7a | How sample size was determined. Method of calculation and relevant parameters with sufficient detail so the calculation can be replicated. Assumptions made about correlations between outcomes of participants from the same cluster | Section 3 (Methods), paragraph 13; study protocol paper |
|  | 7b | When applicable, explanation of any interim analyses and stopping guidelines. | Study protocol paper |
| **Randomisation** | | | |
| Sequence generation | 8a | Method used to generate the random allocation to the sequences of treatments. | Section 3 (Methods), paragraph 10; study protocol paper |
|  | 8b | Type of randomisation; details of any constrained randomisation or stratification, if used. | Section 3 (Methods), paragraph 10; study protocol paper |
| Allocation concealment mechanism | 9 | Specification that allocation was based on clusters; description of any methods used to conceal the allocation from the clusters until after recruitment. | Section 3 (Methods), paragraph 10; study protocol paper |
| Implementation | 10a | Who generated the randomisation schedule, who enrolled clusters, and who assigned clusters to sequences. | Section 3 (Methods), paragraph 10; study protocol paper |
|  | 10b | Mechanism by which individual participants were included in clusters for the purposes of the trial (such as complete enumeration, random sampling; continuous recruitment or ascertainment; or recruitment at a fixed point in time), including who recruited or identified participants. | Section 3 (Methods), paragraph 10; paragraph 12; study protocol paper |
|  | 10c | Whether, from whom and when consent was sought and for what; whether this differed between treatment conditions. | Section 3 (Methods), paragraphs 25-26; study protocol paper |
| Blinding | 11a | If done, who was blinded after assignment to sequences (eg, cluster level participants, individual level participants, those assessing outcomes) and how. | Section 3 (Methods), paragraph 10; study protocol paper |
|  | 11b | If relevant, description of the similarity of treatments. | Not applicable |
| Statistical methods | 12a | Statistical methods used to compare treatment conditions for primary and secondary outcomes including how time effects, clustering and repeated measures were taken into account. | Section 3 (Methods), paragraphs 14-22; study protocol paper |
|  | 12b | Methods for additional analyses, such as subgroup analyses, sensitivity analyses, and adjusted analyses. | Section 3 (Methods), paragraphs 23-24; |
| **Results** | | | |
| Participant flow | 13a | For each treatment condition or allocated sequence, the numbers of clusters and participants who were assessed for eligibility, were randomly assigned, received intended treatments, and were analysed for the primary outcome (see separate SW-CRT flow chart). | Section 4 (Results), paragraph 1, paragraph 3; Figure 3 |
|  | 13b | For each treatment condition or allocated sequence, losses and exclusions for both clusters and participants with reasons. | Section 4 (Results), paragraph 1, paragraph 3; Figure 3 |
| Recruitment | 14a | Dates defining the steps, initiation of intervention, and deviations from planned dates. Dates defining recruitment and follow-up for participants. | S1 Table |
|  | 14b | Why the trial ended or was stopped. | Not applicable |
| Baseline data | 15 | Baseline characteristics for the individual and cluster levels as applicable for each treatment condition or allocated sequence. | Section 4 (Results), paragraph 5;  Table 2 |
| Numbers analysed | 16 | The number of observations and clusters included in each analysis for each treatment condition and whether the analysis was according to the allocated schedule. | Section 4 (Results), paragraph 1, paragraphs 3-5; Figure 3 |
| Outcomes and estimation | 17a | For each primary and secondary outcome, results for each treatment condition, and the estimated effect size and its precision (such as 95% confidence interval); any correlations (or covariances) and time effects estimated in the analysis. | Section 4 (Results), paragraphs 8-9; Table 4; Table 5; Figure 4 |
|  | 17b | For binary outcomes, presentation of both absolute and relative effect sizes is recommended. | Section 4 (Results), paragraphs 8-9; Table 4; Table 5; Figure 4 |
| Ancillary analyses | 18 | Results of any other analyses performed, including subgroup analyses and adjusted analyses, distinguishing prespecified from exploratory. | Section 4 (Results), paragraphs 10-12; Table 6 |
| Harms | 19 | Important harms or unintended effects in each treatment condition (for specific guidance see CONSORT for harms). | Section 4 (Results), paragraph 1 |
| **Discussion** | | | |
| Limitations | 20 | Trial limitations, addressing sources of potential bias, imprecision, and, if relevant, multiplicity of analyses. | Section 5 (Discussion), paragraph 18 |
| Generalisability | 21 | Generalisability (external validity, applicability) of the trial findings. Generalisability to clusters or individual participants, or both (as relevant). | Section 5 (Discussion), paragraphs 11-15, paragraphs 18-23 |
| Interpretation | 22 | Interpretation consistent with results, balancing benefits and harms, and considering other relevant evidence. | Section 5 (Discussion), paragraphs 1-8 |
| **Other information** | | | |
| Registration | 23 | Registration number and name of trial registry. | Section 1 (Abstract); Section 3 (Methods), paragraph 3 |
| Protocol | 24 | Where the full trial protocol can be accessed, if available. | Study protocol paper (attached) |
| Funding | 25 | Sources of funding and other support (such as supply of drugs), and the role of funders. | Section 1 (Abstract); Section 6 (Declarations) Acknowledgements, Funding) |
| Research ethics review | 26 | Whether the study was approved by a research ethics committee, with identification of the review committee(s). Justification for any waiver or modification of informed consent requirements. | Section 3 (Methods) paragraph 25; Section 6 (Declarations) – Ethical Approvals |
