# Supplementary material for: The impact of continuous quality improvement on coverage of antenatal HIV care tests in rural South Africa: Results of a stepped-wedge cluster-randomised controlled implementation trial
Source: PLoS Med. 2020 Oct 7;17(10):e1003150. doi: 10.1371/journal.pmed.1003150 (PMC7540892; doi:10.1371/journal.pmed.1003150)
Supplement: S1 Text — (DOCX) [file pmed.1003150.s002.docx]

**Supplementary File 1**

**The impact of continuous quality improvement on coverage of antenatal HIV care tests in rural South Africa: results of a stepped-wedge cluster-randomised controlled implementation trial**

**This file includes:**

- Additional Methods (description of context, additional statistical methods)
- Figure S1
- Tables S1-S2

## 1.0 Description of context

Viral load (VL) testing and monitoring were entirely paper-based at the study clinics, because at the time of this study electronic antenatal care (ANC) records did not exist. Each clinic usually had one computer, which the clinic data capturer used to enter clinical information from the patient’s medical record onto the national ART electronic monitoring and evaluation (M&E) database, TIER.Net. No clinics in the sub-district had access to internet, and electricity was dependent on the local grid with no back-up generators.

Blood samples from clinics were dispatched daily to Hlabisa Hospital via the hospital transport system. The samples were then centrally dispatched to a tertiary hospital laboratory in Durban. Results were uploaded to the National Health Laboratory Service (NHLS) database. Results were printed and batch-delivered from Hlabisa Hospital to each requesting clinic. The workflow from results delivery to filing in the patient’s medical record varied between clinics. This variation indicates that there may be opportunities to improve the timely delivery of test results, including maternal HIV VL.

Lay counsellors have conducted HIV counselling and testing in South Africa for many years as part of scaling up the antiretroviral therapy (ART) programme. This cadre was introduced as one of the health system’s strategies to cope with chronic health worker shortages. Lay counsellors also conduct adherence counselling to patients on ART [1]. In 2015, The South African National Department of Health (DoH) announced plans to redeploy lay counsellors and evidence from one district in KwaZulu-Natal showed that redeployment led to a reduction in HIV testing [2]. Without lay counsellors, HIV counselling and testing are expected to shift back to nurses.

Source documents used during clinic CQI activities to identify patients eligible for HIV care tests were routine DoH paper-based M&E clinic registers, because HIV VL testing and HIV screening were standard M&E indicators for ANC at study start. Patient medical records (maternity case records, MCRs) were unavailable during clinic CQI meetings as they were retained by pregnant women until delivery.

**Fig S1** summarizes HIV prevalence by age group at the first antenatal visit.

**Tables S1** and **S2** summarize clinic characteristics and staffing.

**
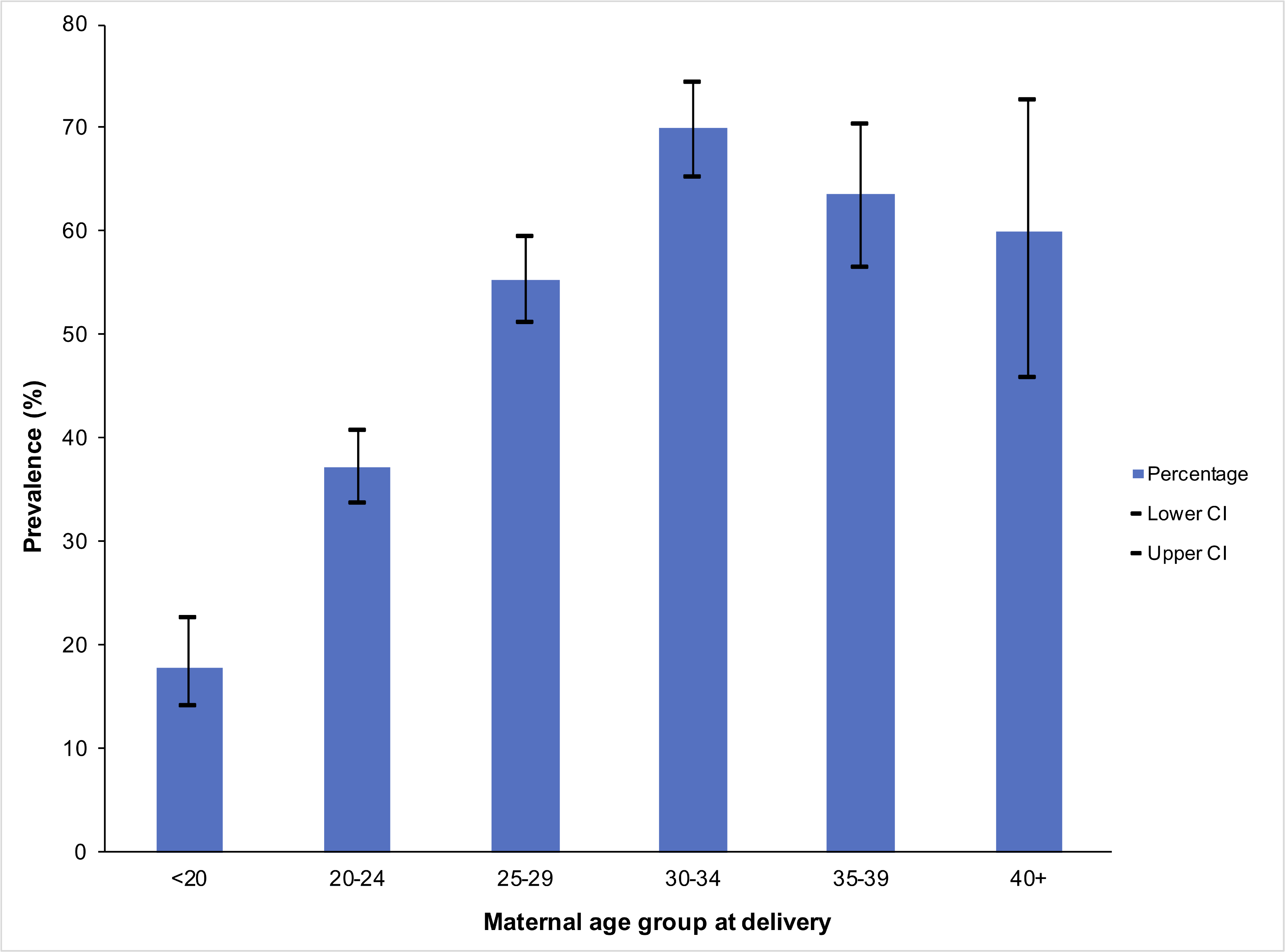
**

**Figure S1. HIV prevalence by age group at first antenatal visit**

**2.0 Trial registration: additional explanatory notes**

The reason for the delay in registration was that the funding for this work was originally obtained for healthcare quality improvement in a district in rural South Africa. The baseline measurement for this study was initially intended to serve as data to inform the selection of approaches for quality improvement. However, during the baseline measurement it became clear that a scientific evaluation of this quality improvement was not only feasible but also desirable for local health workers involved in this effort, as well as for local policy makers, funders and the implementing institution (AHRI). In addition, it became clear during the baseline period that the CQI intervention would have to be rolled out in a stepped fashion because of resource constraints and that randomly selecting the order of roll-out was viewed as the fairest and most acceptable approach by all stakeholders. The trial design and registration thus started during the baseline for the CQI intervention.

**3.0 Additional statistical methods: estimation of cumulative risk of endpoints in CQI unexposed and exposed**

We used the adjusted linear regression model output from our sensitivity analysis as our basis for calculations. We manually computed cumulative risk using the exponential formula “1- exp(-RR*T)” described by Rothman & Greenland [3], where T was the antenatal visit number starting from 1. The fixed effect for time step was a categorical covariate, with a coefficient for each step displayed in relation to the baseline step (Step 0). There were no fixed interaction terms in the model. Given the stepped-wedge design, we calculated cumulative risk in CQI unexposed and exposed using coefficients at Step 3 (when half of the clusters were unexposed and the other half exposed).

For CQI unexposed individuals at visit 1, estimated risk of receiving a VL = coefficient of the intercept (_cons) + coefficient for Step 3.

For CQI exposed individuals at visit 1, estimated risk of receiving a VL = coefficient of the intercept (_cons) + coefficient for Step 3 + coefficient for CQI.

We then proceeded with the exponential formula described above, for up to eight antenatal visits given that the majority of our study population attended at least 4 visits (median six visits).

## Bibliography

1. Bemelmans M, Baert S, Negussie E, Bygrave H, Biot M, Jamet C, et al. Sustaining the future of HIV counselling to reach 90-90-90: a regional country analysis. Journal of the International AIDS Society. 2016;19(1):20751. doi: <https://dx.doi.org/10.7448/IAS.19.1.20751>. PubMed PMID: 27189531;

2. Médecins Sans Frontières (MSF). Bending the curves of the HIV/Tb epidemic in KwaZulu-Natal. Cape Town: MSF, 2016.

3. Greenland S, Rothman KJ. Measures of Occurrence. In: Rothman KJ, Greenland S, editors. Modern Epidemiology. Philadelphia: Lippincott Williams and Wilkins; 2008. p. 33-51.
