# Supplementary material for: The impact of continuous quality improvement on coverage of antenatal HIV care tests in rural South Africa: Results of a stepped-wedge cluster-randomised controlled implementation trial
Source: PLoS Med. 2020 Oct 7;17(10):e1003150. doi: 10.1371/journal.pmed.1003150 (PMC7540892; doi:10.1371/journal.pmed.1003150)
Supplement: S1 Table — (DOCX) [file pmed.1003150.s003.docx]

**Table S1. Characteristics of primary health care facilities listed in the chronological order of randomisation to intervention**

| **Order of rollover to intervention** | **Clinic size** | **Average number of antenatal visits per month during Step 0^*^** | **Setting** | **Intensive intervention phase actual start date** |
| --- | --- | --- | --- | --- |
| 1 | Medium | 99 | Rural | 29 September 2015 |
| 2 | Large | 200 | Urban | 24 November 2015 |
| 3a | Small | 36 | Rural | 26 January 2016 |
| 3b | Very small | 14 | Rural | 28 January 2016 |
| 4 | Large | 151 | Urban | 17 March 2016 |
| 5 | Small | 43 | Rural | 18 May 2016 |
| 6 | Medium | 56 | Rural | 19 July 2016 |

^*^ Step 0 was the period during which all clinics provided baseline data per the stepped-wedge design, i.e. up to 28 September 2015.
