# Supplementary material for: The impact of continuous quality improvement on coverage of antenatal HIV care tests in rural South Africa: Results of a stepped-wedge cluster-randomised controlled implementation trial
Source: PLoS Med. 2020 Oct 7;17(10):e1003150. doi: 10.1371/journal.pmed.1003150 (PMC7540892; doi:10.1371/journal.pmed.1003150)
Supplement: S2 Table — CQI, continuous quality improvement (DOCX) [file pmed.1003150.s004.docx]

**Table S2. Summary of total staffing numbers per clinic identified during situational analyses and recruited clinic CQI team**

| **Numbers** | | | | | | | | |
| --- | --- | --- | --- | --- | --- | --- | --- | --- |
|  | **Operational managers**  (recruited to clinic CQI team) | **Professional nurses**  (recruited to clinic CQI team) | **Enrolled (staff) nurses**  (recruited to clinic CQI team) | **Lay counsellors** (recruited to clinic CQI team) | **Data capturers** (recruited to clinic CQI team) | **Nutritional advisors**  (recruited to clinic CQI team) | **Clinical support officer/**  **administrative clerk**  (recruited to clinic CQI team) | **Total staff**  (total in clinic CQI team) |
| **Clinic 1** | 1 (0) | 6 (2) | 6 (0) | 3 (2) | 1 (1) | 1 (1)* | 1 (1) | 21 (7) |
| **Clinic 2** | 1 (1)* | 20 (2) | 6 (1) | 0 (0) | 2 (2) | 1 (0) | 1 (0) | 31 (6) |
| **Clinic 3a** | 1 (0) | 3 (1)* | 2 (1) | 1 (0) | 1 (1) | 1 (0) | 1 (1) | 10 (4) |
| **Clinic 3b** | 1 (1)* | 2 (1) | 2 (1) | 1 (1) | 1 (1) | 1 (1) | 0 | 8 (6) |
| **Clinic 4** | 1 (1)* | 4 (2) | 5 (1) | 3 (1) | 1 (1) | 1 (0) | 1 (0) | 16 (6) |
| **Clinic 5** | 1^#^ (0) | 4 (2) | 3 (1) | 1 (1) | 1 (1) | 1 (0) | 1 (1) | 12 (6) |
| **Clinic 6** | 1^#^ (1)* | 6 (1) | 5 (1) | 1 (1) | 1 (1) | 1 (1) | 1 (0) | 15 (6) |

CQI, Continuous Quality Improvement

* Person nominated as clinic CQI team leader.

^#^ Acting Operational Manager – this may have been due to retirement or other prolonged absence
