# Supplementary material for: The impact of continuous quality improvement on coverage of antenatal HIV care tests in rural South Africa: Results of a stepped-wedge cluster-randomised controlled implementation trial
Source: PLoS Med. 2020 Oct 7;17(10):e1003150. doi: 10.1371/journal.pmed.1003150 (PMC7540892; doi:10.1371/journal.pmed.1003150)
Supplement: Attachment — Submitted filename: yapa.pdf [file pmed.1003150.s006.pdf]

This paper describes a cluster randomised trial of an intervention to improve antenatal care in KwaZulu Natal.

I was asked for a statistical report and I interpret that to include all aspects of the design and conduct of the study.

Points of detail

Page 6 There are several claims of primacy here and later in the paper. Are these really necessary? Surely the study can stand on its own merits?

Page 8 It is important to know the details about possible delays in registration but I wonder whether they could be in the supplement and just called out here. Readers who are not themselves trialists may be puzzled at the importance given to the delay.

Page 12 The sample size determination is expressed in terms of absolute risk differences (15 percentage points). The main analysis is in terms of relative risk differences (risk ratios) with absolute differences presented as sensitivity analyses (page 13). This is inconsistent. There are good reasons for presenting absolute risk differences despite the issues with linear models for probabilities and I do not see the authors offering a justification for abandoning them as their primary analysis.

Page 13 The two references (authors' references 41 and 42) compare odds ratios and risk ratios but I do not find in either of the references any argument of the relative merits of using the log–binomial model over modified Poisson. It seems to me that the log–binomial has the advantage that is fails to converge in situations when the multivariable risk ratio model is inappropriately leading to impossible predicted values greater than unity. There is some discussion of convergence in Williamson et al. (2013) although some of the software issues there are out of date.

Page 15 I found this a little confusing. I suggest '[. . .] 56.3% had a VL performed of whom 52.4% had a documented result of which [. . .]'

Page 15 We need some explanation of how per visit figures are converted into cumulative. It is not obvious to me how this was done.

Page 19 I find it sad that in 2019 the authors view having collaborators who spoke the vernacular language as a strength when one might have

hoped it had become mandatory. Including it in methods is fine of course.

Page 21 Another unnecessary primacy claim.

Page 22 Good to see the members of the DSMB acknowledged by name (and good to have such a board even if adverse events were not expected).

Page 35, Table 1, sub-section 6 Is there really no evidence on fidelity? That seems a gap in an otherwise comprehensive trial. This is perhaps more of an issue for the referees of the process evaluation paper.

Points of more substance

Clinical importance

If the minimal important clinical difference was 15 percentage points as page 12 suggests then the value of 4.1 reported on page 15 is not important and suggests the trial has in fact failed to detect a meaningful effect despite achieving an arbitrary level of statistical significance. Either I have completely misunderstood or the authors are over–interpreting a small difference.

Treatment of the partially exposed

I am all in favour of intention to treat analyses but is it really wise to account for the 20% partially exposed reported on page 14 as being in the arm their initial clinic was in when they joined the study? In a stepped wedge design people will move from control clinics to treated clinics as the study rolls out even if they attend the same physical clinic.

With any trial of a complex intervention it is hard to know when exactly to assume that intervention took effect. Assuming it is when the intervention started seems quite a strong assumption.

Modelling visits and time

In their analysis section (page 13) the authors state that they used a fixed effect for time step. I do not see how this can be used when the women had in principle many visits spanning the study period and most of them had four

or more. This is also tied to the issue of whether to include a random effect for woman. Clinic level clustering accounts, as far as I can see, for the fact that women within cluster are more similar than women between clusters. It does not account for whether women who were more likely to have a VL monitoring at time i were also more likely to have one at time j.

Summary

I am perplexed by a number of features in the analysis.

Michael Dewey

References

T Williamson, M Eliasziw, and G H Fick. Log–binomial models: exploring failed convergence. *Emerging Themes in Epidemiology*, 10(14), 2013.
