# Supplementary material for: The impact of continuous quality improvement on coverage of antenatal HIV care tests in rural South Africa: Results of a stepped-wedge cluster-randomised controlled implementation trial
Source: PLoS Med. 2020 Oct 7;17(10):e1003150. doi: 10.1371/journal.pmed.1003150 (PMC7540892; doi:10.1371/journal.pmed.1003150)
Supplement: Attachment — Submitted filename: Author response to reviewers_MONARCH_Yapa et al.docx [file pmed.1003150.s007.docx]

04 April 2020

Dear Editor,

**Re: PMEDICINE-D-19-03348**

**Original title:** “Can Continuous Quality Improvement increase coverage of antenatal HIV care tests in rural South Africa? Results of a stepped-wedge cluster-randomised controlled implementation trial

**Revised title:** “The impact of Continuous Quality Improvement on coverage of antenatal HIV care tests in rural South Africa: results of a stepped-wedge cluster-randomised controlled implementation trial”

We thank the editors and reviewers for their insightful and constructive comments. In response, we have substantially revised and improved our manuscript.

Below, we describe in detail how we have responded to each comment by the editor and the reviewers. We show the comments in italics and our responses in normal font. We show the changes that we have made indented and in quotation marks, with line numbers that refer to the revised version of our manuscript.

We believe that our revisions have substantially strengthened our manuscript and increased the value of our results for health policy and practice.

Sincerely,

H. Manisha Yapa, Jan-Walter de Neve, Terusha Chetty, Carina Herbst, Frank Post, Awachana Jiamsakul, Pascal Geldsetzer, Guy Harling, Wendy Dhlomo-Mphatswe, Mosa Moshabela, Philippa Matthews, Osondu Ogbuoji, Frank Tanser, Dickman Gareta, Kobus Herbst, Deenan Pillay, Sally Wyke, Till Bärnighausen

**EDITOR COMMENTS**

*1. In your data statement, rather than specifying “reasonable” requests please adapt the wording to “Requests from researchers who meet the criteria for access to confidential data..” or similar. PLOS’ data policy does not allow authors to be the primary point of contact for data, so please modify the statement to note that contact should be made via the data repository.*

Thank you for this recommendation. We have changed the wording as suggested. It now reads:

“Requests from researchers who meet the criteria for access to fully anonymised	patient level data can be made to the AHRI Research Data Management team via	the AHRI data repository at [www.data.africacentre.ac.za](http://www.data.africacentre.ac.za)”

*2. Please remove the rhetorical question from the title, and add a colon before the study descriptor.*

We have changed the title as you suggest. It now reads:

“The impact of Continuous Quality Improvement on coverage of antenatal HIV care tests in rural South Africa: results of a stepped-wedge cluster-randomised controlled implementation trial“

*3.* *Please add summary demographic information for study participants to your abstract.*

**Author response:** We have added the following summary demographic information: median age, median gestation, and median parity (**line 32**):

“Median age at first booking was 25 [interquartile range, IQR, 21-30] and median gestation at first booking was 19 weeks [IQR 15-24]. Median parity was 1 [IQR 0-2].”

*4. The final sentence of the “methods and findings” subsection of your abstract should summarize the study’s main limitations.*

**Author response:** We have added the following sentence summarizing the study’s main limitations (**line 37**):

“Limitations of our study include that we did not establish impact beyond the duration of this relatively short study and that the transition steps may have been too short to achieve the full potential improvement.”

*5. At line 32 please begin the “conclusions” subsection of your abstract with “In this study we found that…. ” or similar.*

**Author response:** We have edited the “conclusions” subsection of the abstract with the suggested opening phrase; it now reads:

“We found that CQI can be effective at increasing quality of primary care in rural Africa.“

*6. After the abstract, we will need to ask you to add a new and accessible “author summary” section in non-identical prose. You may find it helpful to consult one or two recent research papers published in PLOS Medicine to get a sense of the preferred style.*

**Author response:** We have now added the following “author summary” section in non-identical prose to our manuscript:

**“Why was this study done?**

- Gaps in implementation of evidence-based guidelines can slow progress towards major health systems goals, such as the elimination of mother-to-child transmission of HIV.
- Continuous Quality Improvement (CQI) has the potential to improve service delivery within available resources, and has been successful in achieving this potential in resource-rich settings.
- There is very limited evidence on the impact of CQI in improving health systems in resource-poor primary care.

**What did the researchers do and find?**

- We assigned seven public-sector primary-care clinics in rural South Africa to receive CQI in random sequence.
- The intervention was delivered by a trained team of local CQI mentors who worked collaboratively with clinic health workers.
- The intervention team aimed to address root causes of poor HIV viral load (VL) monitoring among pregnant women living with HIV and repeat HIV testing among pregnant women not living with HIV.
- CQI increased HIV VL monitoring by nearly 40%, but did not affect repeat HIV testing.
- Lay counsellors, who conducted HIV testing and counselling in the clinics, were redeployed during the study period, placing a strain on available human resources for HIV testing.

**What do these findings mean?**

- We found that CQI can be effective in improving quality of primary care in resource-poor communities.
- Improvement in HIV VL monitoring may have been easier to achieve than improvement in repeat HIV testing, because the baseline attainment of this endpoint was far lower.
- Resource shortages, particularly staffing shortages, may have prevented CQI from achieving its full potential.
- Future research should focus on the conditions under which CQI is most likely to be successful.

*7. Please remove the sentence of discussion at line 102, or move this to the discussion section.*

**Author response:** We have removed this sentence.

*8. To your methods section, please add a brief mention of ethics approval and the situation regarding informed consent.*

**Author response:** We have now added this to the end of the Methods section (**line 374**):

*“*Ethics approval for the study was obtained from the University of KwaZulu-Natal Biomedical Research Ethics Committee (reference BE209/14). The ethics approval included a waiver of the requirement for individual consent to access routine clinical data from maternity case records.”

*9. Please avoid making claims of your study being “the first”, and where a claim of primacy is made please add “to our knowledge” or similar.*

**Author response:** We have removed our claim of our study being “the first” from the introduction section. We have changed the sentence:

”Our stepped-wedge cluster RCT aims to establish, for the first time, the effectiveness of CQI on important proximate indicators of the quality of HIV-related ANC in primary care”

to (**line 148**):

”Our stepped-wedge cluster RCT aims to establish the effectiveness of CQI on important proximate indicators of quality of HIV-related ANC in primary care”

We have also changed the following sentence:

“Our stepped-wedge cluster RCT conducted under real-life conditions in rural South Africa showed for the first time that CQI can improve quality of care in primary care in sub Saharan Africa”

to (**line** **501**):

“To our knowledge, this is the first RCT to show that CQI can improve quality of primary care in sub-Saharan Africa.”

*10. We ask you to adapt the first paragraph of the discussion section of your main text so that this provides a summary of the study’s findings.*

**Author response:** We have now adapted the opening statements in the Discussion to summarise the study’s main findings as follows:

“Our stepped-wedge cluster RCT conducted under real-life conditions in rural South Africa showed that CQI improved antenatal HIV VL monitoring, but did not improve repeat HIV testing. To our knowledge, this is the first RCT to show that CQI can improve quality of primary care in sub-Saharan Africa.

The significant improvement in the first of our two primary endpoints – VL monitoring – suggests that CQI may indeed be a good approach to improve quality of primary care. This finding is important for policy: CQI is an attractive approach for resource-poor settings because it works with local health workers and within existing resources. Moreover, CQI is already commonly used to improve quality of care in many resource-poor countries and communities, including sub-Saharan Africa [1-5]. Our trial thus provides evidence for these policies.”

**Comment 11.** Please remove trade marks from your ms.

**Author response:** We have removed the trademark that we previously used in our manuscript. We now use the name of our database system without the trademark (REDCap). We have also added a reference to this database system.

*12. Please revise your reference list so that all entries match journal style. Italics and boldface text should be converted into plain text, for example; a maximum of six author names should be listed followed by “et al”.*

We have carefully edited our reference section to make sure that all references are shown in the PLOS Medicine house style.

*13. Please abbreviate journal names consistently; and add full access information to reference 8.*

**Author response:** To avoid inconsistencies, we have fully spelled out all journal names. Of course, if the editors preferred any other style of referencing journal names, we would be happy to change the references accordingly. We have added full access information to reference 8.

*14. Please provide an update on the status of reference 39.*

**Author response:** Reference 39 (the MONARCH process evaluation) has completed peer review at a health policy journal and we have been invited to revise and resubmit the article.

*15. We ask you to convert the TIDieR checklist into an individual supplementary file, referred to in the main text.*

**Author response:** We have converted the TIDieR checklist into an individual supplementary file (Supplementary File 3). We refer to the TIDieR checklist in the main text (**line 201**).

*16. Please also convert your CONSORT checklist into an individual supplementary file, referred to in the methods section of your main text. In the checklist, individual items should be referred to by section (e.g. Methods) and paragraph number rather than by page or line numbers, as the latter generally change in the event of publication.*

**Author response:** We have converted the CONSORT checklist into an individual supplementary file.

In the checklist, we now refer to individual items by section and paragraph number.

We refer to the CONSORT checklist in the methods section of our main text (**line 196**).

**REVIEWER COMMENTS**

**Reviewer 1**

*1.0. The process of evaluation of care (CQI) described here is extremely interesting and the authors/ actors of this work should be congratulated for this approach. As a clinician, non-expert in this type of approach I have only a few “naïve” questions.*

**Author response:** We are grateful to Reviewer 1 for the positive and constructive feedback.

*1.1. The quantitative results of VL at baseline were satisfying. Are the additional VL made thanks to the CQI at the same level?*

**Author response:** Yes, according to our causal effect size estimates, additional VL are made thanks to CQI at all levels of baseline attainment (i.e., attainment in the control arm of this trial). Over a woman’s pregnancy, the CQI intervention will on average increase the number of VL tests by 12 percentage points. We have improved our discussion of these results (**lines 515-523**):

"Policy makers should thus consider our relative effect size in the context of their health system’s achievement in measuring this critical indicator of success in interrupting transmission of HIV from mother to child. In absolute terms, our effect size was relatively small on a per-visit basis, about 4 percentage points. However, it is well recognised that multiple ANC visits are required for maternal and neonatal health [6]. For the median number of six ANC visits in this community, we estimate a cumulative absolute effect size of about 12 percentage points. This effect size is close to the threshold of 15 percentage points for clinically meaningful effects that we used in our power calculations.”

We have also removed the “baseline” (Step 0) VL monitoring estimates from the Methods section, because this estimate only describes average attainment before the start of the CQI intervention and is thus misleading given that the VL attainment in Step 0 forms only a small part of the entire control arm. We emphasize the VL attainment in the full control arm of our trial, which is the correct comparator for the effect size (**lines 447-452**):

“With a median of six ANC visits per pregnancy in this study, the absolute effect size per visit translates to a cumulative probability of receiving a VL test in pregnancy of 58% in the intervention arm compared to 46% in the control arm. With the recent WHO recommendations for at least eight ANC visits [6], these values are equivalent to a cumulative probability of receiving a VL test of 69% in the intervention arm compared to 57% in the control arm.”

*1.2. Why not choose the most important endpoint: “child infection” (or least PCR positive at 6 weeks for example)?*

**Author response:** Ultimately, the most important endpoints are indeed health outcomes, including “child infection” and even child mortality. We did not choose the endpoint “child infection” for this implementation trial because the CQI intervention directly acts on quality of care measures rather than on health outcomes and because it would have been very difficult to sufficiently power the trial for this endpoint. However, because our primary endpoints are necessary steps for prevention of mother-to-child transmission of HIV and child infection, we know that improvement in their attainment is likely to also improve downstream health outcomes. We have added text and improved our previous explanation of our choice of endpoints:

In the introduction section (**lines 107-132**):

“To date, there have only been two randomized controlled trials (RCTs) that have tested whether CQI improves quality of antenatal care in the primary care	systems in sub-Saharan Africa, one in Malawi [7] and the other one in Nigeria [8]. Both trials found CQI to be ineffective [7, 8]. One possible reason for the lack of effectiveness in these two trials is that the investigators evaluated CQI in terms of health outcomes – neonatal and perinatal mortality rates in the study in Malawi [7] – and 6-month postpartum retention in the study in Nigeria [8]. These endpoints are important and they are rigorously assessed in the two studies. However, these endpoints are quite distal from CQI activities, which took place in ANC in primary care clinics. The variability of these endpoints is thus likely largely a function of factors outside the sphere of influence of CQI, such as	emergency care availability, road infrastructure, and mothers’ educational attainment.

For our RCT of CQI effectiveness, we thus decided to use two primary endpoints that are closely and directly linked to the CQI activities. Our trial took place in an HIV hyperendemic community in rural South Africa, where ANC in primary care fulfils two important HIV care-related functions: to ensure that all pregnant women living with HIV are – first – identified and – second – successfully treated with antiretroviral therapy (ART). We choose measures that capture the procedural quality of care of these two key ANC functions (VL monitoring and repeat HIV testing) as primary endpoints — rather than health outcomes — for the following reasons: both endpoints are important measures of ANC quality and are necessary conditions for preventing mother-to-child transmission of HIV (MTCT) [9, 10]. Ultimately, the purpose of healthcare is to improve health outcomes. Implementation science, however, focuses on the improvement of processes whose intermediate outcomes such as testing coverage, can be directly influenced by changes to health services and are known to be strong determinants of health outcomes [11].”

We further explain in the discussion section that one likely reason why two previous studies of CQI in ANC in sub-Saharan Africa did not find significant effects was that the investigators choose health outcomes as their primary endpoints (**lines 586-592**):

“The two other trials of CQI in primary care in sub-Saharan Africa did not find significant effects, likely due to selection of endpoints that were relatively distal to CQI activities. As alluded to previously, the variability of these endpoints — neonatal and perinatal mortality in Malawi [7], and a distal healthcare utilization outcome, postpartum retention in Nigeria [8]— may be a function of factors outside the sphere of influence of CQI. Our endpoints were more proximate to CQI thereby making it relatively easier to demonstrate a direct CQI effect.”

And **lines 726-730**:

“While our quality of care measures are good choices to test the effectiveness of a	CQI intervention, the ultimate purpose of healthcare is to reduce morbidity and	mortality. Future mathematical modelling studies should estimate the impact of CQI	on health outcomes that could be achieved given the effect sizes we measured in	this study and local estimates of VL monitoring in different communities in sub-	Saharan Africa.”

And **lines 715-725**:

“Outcome measures that lie on plausible causal pathways from an exposure to an outcome can be used to empirically confirm mechanistic hypotheses. CQI is an intervention that can work its way to outcomes through multiple pathways, because it empowers local health workers to identify and implement those health systems improvements and innovations that are most promising in the particular contexts in which they work. In this case – and other complex interventions – local health workers can identify likely mechanisms connecting an intervention to outcomes. In the case of HIV VL monitoring and repeat HIV testing, proximal endpoints lying on the causal pathways from CQI could include availability of test kits, presence of systems for patient tracking and tracing , health worker motivation to test, [12] and patient demand for testing.”

**Concerning the CQI itself:**

*1.3.* *It is stated that the teams have welcomed the CQI procedure. Did the level of satisfaction remain similar after the CQI?*

**Author response:** We thank the reviewer for this important question. We have added the following text to address it:

In the Results section (**lines 388-394**):

“The health workers appreciated the CQI intervention throughout the study period. For instance, the CRH team observed that the clinic health workers who participated in the CQI intervention were enthusiastic about their increased capacity to improve quality of services. Several clinic health workers expressed an interest in further training and mentoring on quality improvement tools and approaches. Finally, clinic health workers urged the CRH team to continue to work with them on quality improvement beyond the end of this study.”

And in the Discussion section (**lines 602-605**):

“Based on our observations and process evaluation, health workers maintained positive attitudes towards CQI and our CQI mentors during the study. In particular, health workers were enthusiastic to learn CQI tools and improve quality of services, and were keen to continue working with the CRH team. ”

*1.4. It would be interesting to specify in more detail the time and human resources required for these interventions, in order to appreciate the feasibility in other contexts.*

**Author response:** We have added a detailed discussion of the human resources required for the CQI intervention. We have further added a discussion of the feasibility in other contexts and scalability (**lines** **625-658**):

“The key resource needed to implement CQI is CQI mentors’ and clinic health workers’ time. CQI mentors typically spent several hours per week training and supporting clinic health workers during the intensive phase of CQI; during the maintenance phase, the mentors spent substantially less time interacting with the clinic health workers, typically several hours per month. The training sessions at the very beginning of each step and the action learning sessions each required an entire day. CQI activities required time to (i) identify drivers of poor VL monitoring and repeat HIV testing, (ii) conduct group meetings to design clinic-specific solutions, (iii) implement those solutions (e.g. different methods to track women eligible for HIV care tests), and (iv) use clinic-based real-time data sources to test whether their solutions worked towards target testing rates.

CQI mentors who are adaptable and relate professionally and culturally to health workers at target health facilities, may be more successful at engaging health workers to implement CQI. Mentors must be adept at generating descriptive data, and ideally familiar with implementing the desired change in practice. Obtaining basic CQI certification may take around 18 hours (online)[13] to three days [14] of coursework.

In addition to the CQI mentors’ time and the health workers’ time, a so-called Improvement Advisor – a more highly qualified health professional than the CQI mentors with the ability to coach and lead the CQI mentors – is an integral part of the team to oversee success. In our experience, the Improvement Advisor would need to invest around 20% of their time; in other settings this would depend on the needs of the CQI mentors and participating clinics. Thus, the time – and financial – costs of the intervention were overall low. Feasibility and scalability of CQI will, however, not only depend on resource requirements but also on clinic health workers’ motivation and mindset. CQI requires changes in individual behaviour and institutional practice, which in turn are driven by job satisfaction, ambition, experience and local workplace culture and constraints [50].

In our study, the CQI mentors typically held CQI meetings 2-3 times per week during the intensive two-month step. For routine implementation at larger scale, alternative meeting frequencies may also work. For instance, it may be more practicable to conduct visits less frequently (e.g. once per week) but over a longer period of time (e.g. half a year). The optimal frequency of mentor visits will likely depend on how many clinic health workers are available for CQI activities and whether the clinic activities that the CQI chose for improvement require additional support.”

*1.5. Were the interventions homogeneous between the centers and during the study period? Can we imagine a sort of “CQI of the CQI”?*

**Author response:** We thank the reviewer for these two important and interesting questions. We have added the following text to our discussion section (**lines 606-624**):

“CQI is a systematic process of identifying problems and devising, testing and revising potential solutions to quality shortcomings. The different management techniques used during this process are highly standardized and time-tested, including process mapping, Fishbone diagrams and PDSA cycles. Deviations from the overall process and these individual techniques — by the CQI mentors implementing them, or by clinic health workers utilising them — would imply low fidelity. Based on our observations, CQI mentors implemented the CQI intervention with high fidelity. However, clinic health workers’ implementation of the CQI process and techniques was of somewhat lower fidelity. Clinic health workers could not always participate in CQI meetings and failed to complete some of the CQI tasks, despite their enthusiasm [12]. The reasons for these deviations were mostly rooted in structural capacity constraints in this rural South African health system, including staffing shortages, rapid staff turnover, and competing clinical commitments [15].

The CQI process and management techniques are intended to be the same across clinics and communities; the solutions that they identify, test and revise are intended to be locally adapted and will thus differ across contexts. Our process evaluation accompanying this RCT revealed that the solutions that each clinic identified and implemented did indeed differ, even though all participating clinics were located in the same district in rural KwaZulu-Natal [12].

*1.6. After this procedure, can we consider a simplified procedure of CQI to be more routinely implemented (at least for this specific question of HIV in ANC)?*

**Author response**: We have added a discussion about the routine implementation of CQI to our discussion section (**lines** **652-658**):

“In our study, the CQI mentors typically held CQI meetings 2-3 times per week during the intensive two-month step. For routine implementation at larger scale, alternative meeting frequencies may also work. For instance, it may be more practicable to conduct visits less frequently (e.g. once per week) but over a longer period of time (e.g. half a year). The optimal frequency of mentor visits will likely depend on how many clinic health workers are available for CQI activities and whether the clinic activities that the CQI chose for improvement require additional support.

And **lines** **699-704**:

“Improving clinical processes to achieve desired health systems and health outcomes requires changes in practice (behavioural changes), which depend on individual motivation, job satisfaction, and clinical knowledge and context [16]. It is also unclear as to how far clinic health workers ‘normalise’ CQI into their practice once the CQI mentors have completed an engagement, particularly given the human resource shortages and staff turnover we are aware of.”

*1.7. Did CQI related improvement of care for HIV women, have an impact (favourable or deleterious or neutral) on other activities of ANC (outside HIV care)?*

**Author response:** We thank the reviewer for this important point. We have added the following text to our results section (**lines** **384-387**):

“The clinic nurses and local DoH were continuous partners in this intervention throughout the study duration. There were no adverse events, complaints or other deleterious effects of the CQI intervention reported.”

We have also added the following text to our discussion section (**lines 707-714**):

“An important question for future research is whether this CQI intervention ‘spilled-over’ to ANC activities outside HIV care and to primary care outside ANC. Such spill-over effects could be positive or negative. On the one hand, the clinic health workers trained in CQI may have applied their new skills to other clinical services, resulting in further quality improvements. On the other hand, the health workers invested time and energy in this CQI and may, as a result, have neglected clinical services outside the scope of this intervention. In future research, we plan to measure the effects of CQI on a broader set of endpoints, which will capture the quality of a wide range of ANC functions.”

**Reviewer 2**

2.1. Page 6. There are several claims of primacy here and later in the paper. Are these really necessary? Surely the study can stand on its own merits?

**Author response:** We have removed our claim of our study being “the first” from the introduction section. We have changed the sentence:

”Our stepped-wedge cluster RCT aims to establish, for the first time, the effectiveness of CQI on important proximate indicators of the quality of HIV-related ANC in primary care”

to (**line 148**):

”Our stepped-wedge cluster RCT aims to establish the effectiveness of CQI on important proximate indicators of the quality of HIV-related ANC in primary care”

We have also changed the following sentence:

“Our stepped-wedge cluster RCT conducted under real-life conditions in rural South Africa showed for the first time that CQI can improve quality of care in primary care in sub Saharan Africa”

to (**line 501**):

“To our knowledge, this is the first RCT to show that CQI can improve quality of primary care in sub-Saharan Africa.”

*2.2. Page 8. It is important to know the details about possible delays in registration but I wonder whether they could be in the supplement and just called out here. Readers who are not themselves trialists may be puzzled at the importance given to the delay.*

**Author response:** We agree and have now summarised the delay in the Methods section and moved details to the Supplementary Material as suggested.

We have changed the text from:

“Trial registration occurred after the baseline and the first step of this eight-step stepped-wedge RCT, on 10 December 2015. The reason for this registration timing was that the funding for this work was originally obtained for healthcare quality improvement in a district in rural South Africa. The baseline measurement for this study was initially intended to serve as data to inform the selection of approaches for quality improvement. However, during the baseline measurement it became clear that a scientific evaluation of this quality improvement was not only feasible but also desirable for local healthcare providers involved in this effort, as well as for local policy makers, funders and the implementing institution (AHRI). In addition, it became clear during the baseline period that the CQI intervention would have to be rolled out in a stepped fashion because of resource constraints and that randomly selecting the order of roll-out was viewed as the fairest and most acceptable approach by all stakeholders. The trial design and registration thus started during the baseline for the CQI intervention. The stepped-wedge design was selected for both pragmatic and ethical reason [17]. The description of our results follows the CONSORT extensions for stepped-wedge trials [18] (**Supplementary File 2**).”

To (**lines** **190-197**):

“Trial registration occurred after the baseline and the first step of this eight-step stepped-wedge RCT, on 10 December 2015. The reason for this timing was that it became clear during the baseline that a rigorous scientific evaluation of the CQI intervention would be feasible and desirable for both government and implementing partners (**Supplementary File 1**). The stepped-wedge design was selected for both pragmatic and ethical reasons [17]. The description of our results follows the 2018 Consolidated Standards of Reporting Trials (CONSORT) extension for stepped-wedge RCTs [18] (**Supplementary File 2**).”

*2.3. Page 12. The sample size determination is expressed in terms of absolute risk differences (15 percentage points). The main analysis is in terms of relative risk differences (risk ratios) with absolute differences presented as sensitivity analyses (page 13). This is inconsistent. There are good reasons for presenting absolute risk differences despite the issues with linear models for probabilities and I do not see the authors offering a justification for abandoning them as their primary analysis.*

**Author response:** We have now elevated the absolute difference from a sensitivity analysis to a primary analysis in our manuscript and emphasize and interpret both the relative and absolute measures of effects. The 2018 extension of the CONSORT statement does indeed recommend presenting both the relative and the absolute effect sizes, because of their complementary advantages.

We now describe this methods choice in the revised statistical methods and emphasize the measurement of both relative and absolute effect sizes in several places (**lines** **318-372**):

“Primary analyses: The 2018 extension of the CONSORT statement for the stepped-	wedge cluster RCT states that “in addition to reporting a relative measure of the	effect of the intervention it can be helpful to report an absolute measure of the effect”.	The reasons for this recommendation is that “relative measures of the effects are	often more stable across different populations” [18] and thus a more useful measure	for policy makers considering adopting an intervention that has generated a certain	effect, while “absolute measures of effects are more easily understood” [18]. We	follow this recommendation and report and interpret both the relative and absolute	effect sizes as our primary analyses.

For the primary analysis, we estimated the relative effect sizes using modified Poisson mixed effects generalised linear regression models. Modified Poisson regression has become a standard for the analysis of RCTs with binary outcomes [19, 20], because it has advantages over both logistic and log-binomial regression models [19, 21]. One advantage of modified Poisson regression is that it directly generates risk ratios rather than the odds ratios that logistic regression generates. As relative measures of effect, risk ratios have advantages over odds ratios because they are easier to interpret and collapsible [22-24].

Another advantage of modified Poisson regression is that it does not suffer from the convergence problems that commonly arise with another approach to estimate risk ratios, log-binomial regression [25-27]. In addition, modified Poisson regression models are more robust to model misspecification than log-binomial regression models [28]. A disadvantage of modified Poisson regression is that it can produce predicted probabilities greater than unity. However, several simulation studies have shown that modified Poisson regression provides risk ratio estimates equivalent to regression models that cannot produce such predicted probabilities, such as log-binomial regression [25, 29, 30]. Modified Poisson regression models are thus a good choice for estimating risk ratios in RCTs [20]. To measure absolute effect sizes, we used mixed effects linear probability regression models.

We use three models to estimate the effect of the CQI intervention. First, we used the standard Hussey and Hughes model, which includes clinic random effects and time step fixed effects (Model 1 in **Table 3**) [31]. Second, we used an extension to this model with a random clinic-time step interaction term (Model 2 in **Table 3**). This extension is recommended by Hemming et al. [32], because it allows secular time trends to vary randomly by clinic. Third, we extended the standard Hussey and Hughes model with individual random effects nested within clinic random effects (Model 3 in **Table 3**). This model accounts not only for clustering of outcomes by clinic but also for clustering of outcomes within individual women across time steps. In all models, we used cluster robust standard errors to further adjust for clustering and any model misspecification in all regressions [19, 20].

In addition to the per-visit effect sizes described above, we also computed the cumulative absolute probabilities of attaining our endpoints. For this purpose, we used the per-visit absolute effect sizes measured in Model 2 (**Table 4)** and applied the exponential formula to estimate the cumulative probabilities across the average number of visits during a pregnancy [33].

Sensitivity analyses: For both of our primary analyses – estimating relative and absolute risk – we also ran regressions with additional control variables. We added (i) maternal age and parity; (ii) gestation at each ANC visit and total number of ANC visits attended in pregnancy; and (iii) maternal age, parity, gestation at each ANC visit, and total number of ANC visits.

Analysis of effect heterogeneity: We measured CQI effect heterogeneity by duration of CQI exposure, using the same mixed effects regression models as in the primary analyses but replacing the fixed effect for overall CQI exposure with fixed effects for CQI exposure for each time step since rollover to CQI.

We used Stata version 15.0 (StataCorp. 2017. College Station, TX: StataCorp LLC) for all statistical analyses.

We have also revised our Results section (**lines** **440-495**):

“*Effect of CQI on HIV care tests*

Primary analyses – pregnant women living with HIV: Women exposed to CQI were significantly more likely to receive a VL test: The relative effect sizes in our three primary analyses were essentially equal, ranging from 1.38 to 1.39 (**Table 3, Figure 4**), and were all highly significant (all *p*<0.001). Similarly, the absolute effect sizes of receiving a VL test per ANC visit were very similar in all three primary analyses, 3.9 to 4.1 percentage points (**Table 4**) in the intervention compared to the control arm. With a median of six ANC visits per pregnancy in this study, the absolute effect size per visit translates to a cumulative probability of receiving a VL test in pregnancy of 58% in the intervention arm, compared to 46% in the control arm. With the recent WHO recommendations for at least eight ANC visits [6], these values are equivalent to a cumulative probability of receiving a VL test of 69% in the intervention arm, compared to 57% in the control arm.

Primary analysis – pregnant women not living with HIV: CQI did not have a statistically significant effect on repeat HIV testing in any of our three primary analyses. The RRs ranged from 1.00 to 1.01 and the corresponding p-values ranged from 0.877 to 0.958 (**Table 3** and **Figure 4**). These numbers are equivalent to an absolute change in the probability of receiving a repeat HIV test per ANC visit of 0.0 to 0.3 percentage points (all *p*≥0.758, **Table 4**). With a median of six ANC visits in this study, this effect size per visit translates to a 59-60% cumulative probability of receiving a repeat HIV test during pregnancy in both intervention and control arms. With the WHO-recommended eight ANC visits, these numbers translate to 69-70% cumulative probability in both arms.

Sensitivity analyses: When we adjusted for additional covariates, all effect size estimates remained essentially the same as in the primary analyses. RRs for HIV VL monitoring ranged from 1.36 to 1.37 (all *p*<0.001) with adjustments for maternal age and parity; 1.39 to 1.40 (*p*-values <0.001 to 0.001) with adjustments for gestation at each visit and total number of ANC visits; and 1.35 to 1.36 (*p*-values 0.005 to 0.009) with adjustments for all four covariates. The absolute effect sizes for VL monitoring per ANC visit were 3.7 to 4.0 percentage points (all *p*<0.001) with adjustments for maternal age and parity; 4.4 to 4.6 percentage points (all *p*<0.001) with adjustments for gestation at each visit and total number of ANC visits; and 4.2 to 4.4 percentage points (all *p*≤0.008) with adjustments for all four covariates.

RRs for repeat HIV testing ranged from 1.00 to 1.01 (all *p* ≥0.871) with adjustments for maternal age and parity; 1.08 (all *p*≥0.525) with adjustments for gestation at each visit and total number of ANC visits; and 1.08 (all *p*≥0.504) with adjustments for all four covariates. The absolute effect sizes for repeat HIV testing per ANC visit ranged from 0.0 to 0.3 percentage points (all *p*≥0.757) with adjustments for maternal age and parity; 1.1 to 1.3 percentage points (all *p*≥0.457) with adjustments for gestation at each visit and total number of ANC visits; and 1.1 to 1.3 percentage points (all *p*≥ 0.445) with adjustments for all four covariates.

CQI effect heterogeneity: There was variability in CQI effect on VL monitoring with time since rollover to the intervention, in all three mixed effects models. Immediately after rolling over to the intervention, the relative effect size was 1.17 (*p*=0.071) in Model 1. After two months of CQI exposure (lag of one step) and four months (lag of two steps), the relative effect sizes were 1.42 (*p*=0.021) and 1.45 (*p*=0.015) respectively. There was no significant CQI effect beyond six months of exposure (**Table 5**). Similarly, the absolute effect sizes varied with the same durations of exposure to CQI in Model 1: 1.7 percentage points (*p*=0.070) with immediate exposure, 4.4 percentage points (*p*=0.038) after two months of exposure, 4.9 percentage points (*p*=0.035) after four months of exposure and no significant change beyond six months of exposure (**Table 5**). The results were essentially the same with Models 2 and 3 for relative and absolute effect sizes. There was no heterogeneity in CQI effect on repeat HIV testing by duration of CQI exposure in either relative or absolute measures (**Table 5**).”

.

*2.4. Page 13. The two references (authors’ references 41 and 42) compare odds ratios and risk ratios but I do not find in either of the references any argument of the relative merits of using the log-binomial model over modified Poisson. It seems to me that the log-binomial model has the advantage that it fails to converge in situations when the multivariable risk ratio model is inappropriately leading to impossible predicted values greater than unity. There is some discussion of convergence in Williamson et al (2013) although some of the software issues there are out of date.*

**Author response:** Thank you for this comment. We have substantially improved our argument of the relative merits of using the modified Poisson model over the log-binomial model. We have also added references to support our argument (**lines 327-345**).

“For the primary analysis, we estimated the relative effect sizes using modified Poisson mixed effects generalised linear regression models. Modified Poisson regression has become a standard for the analysis of RCTs with binary outcomes [19, 20], because it has advantages over logistic and log-binomial models [19, 21]. One advantage of modified Poisson regression is that it directly generates risk ratios rather than the odds ratios that logistic regression generates. As relative measures of effect, risk ratios have advantages over odds ratios because they are easier to interpret and collapsible [22, 23, 34].

Another advantage of modified Poisson regression is that it does not suffer from the convergence problems that commonly arise with another approach to estimate risk ratios, log-binomial regression [25-27]. In addition, modified Poisson regression models are more robust to model misspecification than log-binomial regression models [28]. A disadvantage of modified Poisson regression is that it can produce predicted probabilities greater than unity. However, several simulation studies have shown that modified Poisson regression provides risk ratio estimates equivalent to regression models that cannot produce such predicted probabilities, such as log-binomial regression [25, 29, 30, 35]. Modified Poisson regression models are thus a good choice for estimating risk ratios in RCTs [20]. To measure absolute effect sizes, we used mixed effects linear probability regression models.”

*2.5. Page 15. I found this a little confusing. I suggest ‘[…] 56.3% had a VL performed of whom 52.4% had a document result of which […]’.*

**Author response:** Thank you for this comment. We have improved this section. It now reads (**lines** **426-430**):

“At any time in pregnancy, 93.8% of women living with HIV had a documented ART prescription and 56.3% had received a VL test. Among those women who had received a VL test, 52.4% had a documented result of which 85.2% were <200 copies/mL (**Table 2**).”

*2.6. We need some explanation of how per visit figures are converted into cumulative. It is not obvious to me how this was done.*

**Author response:** We have improved our explanation of how we converted the per visit figures into cumulative ones.

In the Methods section (**lines** **357-361**) we have improved our description as follows:

“In addition to the per-visit effect sizes described above, we also computed the	cumulative absolute probabilities of attaining our endpoints. For this purpose, we	used the per-visit absolute effect sizes measured in Model 2 (**Table 4)** and applied	the exponential formula over all visits during an average a pregnancy [33].”

*2.7. I find it sad that in 2019 the authors view having collaborators who spoke the vernacular language as a strength when one might have hoped it had become mandatory. Including it in methods is fine of course.*

**Author response:** We agree. Research partners who speak the vernacular should be the normal state and not a particular strength. This is indeed the case in our study. Here, we had merely highlighted that the CQI intervention team spoke isiZulu, which is clearly a requirement for the intervention to succeed. We have improved our discussion section (**lines** **669-674**):

“Our CQI implementation team of CQI mentors, Improvement Advisor, data manager and scientific partners were from the University of KwaZulu-Natal, had extensive experience implementing CQI elsewhere in South Africa, and spoke the vernacular language, isiZulu. The research was co-led by South African researchers, whilst most researchers in the wider scientific team have worked in rural South Africa for many years.”

*2.8. Page 21. Another unnecessary primacy claim.*

**Author response:**

We have now changed the following sentence:

“Our stepped-wedge cluster RCT conducted under real-life conditions in rural South Africa showed for the first time that CQI can improve quality of care in primary care in sub Saharan Africa”

to (**line** **501**):

“To our knowledge, this is the first RCT to show that CQI can improve quality of primary care in sub-Saharan Africa.”

*2.9. Page 22. Good to see the members of the DSMB acknowledged by name (and good to have such a board even if adverse events were not expected).*

**Author response:** We thank Reviewer 2 for his positive feedback.

*2.10. Table 1, sub-section 6. Is there really no evidence on fidelity? That seems a gap in an otherwise comprehensive trial. This is perhaps more of an issue for the referees of the process evaluation paper.*

**Author response:** We agree with Reviewer 2 on the importance of evaluating intervention fidelity of a complex intervention such as CQI, because fidelity determines reproducibility and scalability of the intervention. We have added the following text to the discussion section in the main manuscript (**lines 606-624**):

“CQI is a systematic process of identifying problems and devising, testing and revising potential solutions to quality shortcomings. The different management techniques used during this process are highly standardized and time-tested, including process mapping, Fishbone diagrams and PDSA cycles. Deviations from the overall process and these individual techniques — by the CQI mentors implementing them, or by clinic health workers utilising them — would imply low fidelity. Based on our observations, CQI mentors implemented the CQI intervention with high fidelity. However, clinic health workers’ implementation of the CQI process and techniques was of somewhat lower fidelity. Clinic health workers could not always participate in CQI meetings and failed to complete some of the CQI tasks, despite their enthusiasm [12]. The reasons for these deviations were mostly rooted in structural capacity constraints in this rural South African health system, including staffing shortages, rapid staff turnover, and competing clinical commitment [15].

The CQI process and management techniques are intended to be the same across clinics and communities; the solutions that they identify, test and revise are intended to be locally adapted and will thus differ across contexts. Our process evaluation accompanying this RCT revealed that the solutions that each clinic identified and implemented did indeed differ, even though all participating clinics were located in the same district in rural KwaZulu-Natal [12].”

We also describe several key fidelity factors in **Supplementary file 3** (originally Table 1) sub-section 12 (“How well, actual”).

**Points of more substance:**

*2.11. Clinical importance. If the minimal important clinical difference was 15 percentage points as page 12 suggests then the value of 4.1 reported on page 15 is not important and suggests the trial has in fact failed to detect a meaningful effect despite achieving an arbitrary level of statistical significance. Either I have completely misunderstood or the authors are over-interpreting a small difference.*

**Author response:** The primary effect size that we measure using the data from our trial is a per-visit effect size. We established the minimal detectable difference in discussions with local health policy makers and community stakeholders. In these discussions, we used the construct of a pregnant woman and the probability that she would receive the needed HIV-related tests that are our primary endpoints captured over the course of her pregnancy. This approach to thinking about HIV-related testing in pregnancy was a more natural and better approach to discussing testing probabilities than an equivalent per-visit effect size. The per-pregnancy effect size based on our per-visit results is close to the minimal detectable difference in our power calculations (12% vs. 15%).

We have improved our description of our sample size calculation (**lines 286-291**):

“If we enrolled a total of 1260 pregnant women (i.e., 630 women living with HIV and 630 women not living with HIV), we estimated 80% power to detect at least a 15-percentage point increase in our two primary endpoints at the 5% significance [17]. This minimum detectable difference	over the course of a pregnancy was identified as meaningful for health policy and clinical practice.”

We have also added the following text to our Discussion section to better interpret our effect sizes in their relevance for health policy and clinical practice (**lines** **510-523**):

“Both relative and absolute effect sizes are important for policy. We find a large relative effect of the CQI intervention on VL monitoring: CQI caused an almost 40% increase in VL monitoring. Relative effect sizes are commonly more stable across populations with different baseline attainments of an endpoint [36]. VL monitoring varies widely across ANC and HIV treatment programmes in sub-Saharan Africa [37, 38]. Policy makers should thus consider our relative effect size in the context of their health system’s achievement in measuring this critical indicator of success in interrupting transmission of HIV from mother to child. In absolute terms, our effect size was relative small on a per visit basis, about 4 percentage points. However, it is well recognised that multiple ANC visits are required for maternal and neonatal health [6].For the median number of six ANC visits in this community, we estimate a cumulative absolute effect size of about 12 percentage points. This effect size is close	to the threshold of 15 percentage points for clinically meaningful effects that we used in our power calculations.”

*2.12. Treatment of the partially exposed. I am all in favour of intention to treat analyses but is it really wise to account for the 20% partially exposed reported on page 14 as being in the arm their initial clinic was in when they joined the study? In a stepped wedge design people will move from control clinics to treated clinics as the study rolls out even if they attend the same physical clinic.*

**Author response:** We did indeed allow individual women’s exposure status to vary across their ANC visits, according to the exposure status of ‘their’ clinic at the time of a particular visit. Our intention to treat assignment was to the clinic at which a woman first attended ANC. If this clinic changed exposure status between two or more of a woman’s visits, the woman’s exposure status changed accordingly. We have improved our description in the methods section, explaining the exposure assignment of partially exposed women (**lines** **292-302**).

“We performed Intent-to-Treat (ITT) analyses based on the clinic attended at the first	antenatal booking visit – individuals declared their ‘intention’ to attend that same facility	for the remainder of pregnancy. Although it is well established that the AHRI	surveillance population is mobile [31], ITT assumes exposure to a single clinic for the	entire duration of pregnancy regardless of actual attendance elsewhere. All	participants were assigned CQI exposure status at each ANC visit (by the actual date	of that visit) according to the exposure status of their clinic at that time. Participants	whose assigned clinic rolled over to CQI during their antenatal care thus had one or	more initial ANC visits that were CQI unexposed and one or more later visits that were	CQI exposed. The beginning of each step (CQI rollover date) was defined as the date	of the first actual CQI intervention visit in the randomised cluster.”

*2.13. With any trial of a complex intervention it is hard to know when exactly to assume that intervention took effect. Assuming it is when the intervention started seems quite a strong assumption.*

**Author response:** We agree with the reviewer. We have added an analysis of effect heterogeneity over time since intervention start. This analysis has added substantial value to our paper, because it reveals a strong and plausible pattern of effect development following the start of the intervention: initially the effect size is modest; it then increases; and, finally, it decreases to baseline levels (**Table 5**).

**Table 5. CQI effect heterogeneity by time since rollover to CQI**

| Variable | | | VL monitoring  *n*=6397 | | Repeat HIV testing  *n*=6815 | |
| --- | --- | --- | --- | --- | --- | --- |
|  |  |  | **Model 1**  **RR**  **(95% CI)**  ***p*-value** | **Model 1**  **Absolute RD**  **(95% CI)**  ***p*-value** | **Model 1**  **RR**  **(95% CI)**  ***p*-value** | **Model 1**  **Absolute RD**  **(95% CI)**  ***p*-value** |
| CQI effect: time steps since CQI rollover | | |  |  |  |  |
|  | Immediate | | 1.17  (0.99 – 1.39)  0.071 | 0.017  (-0.001 — 0.036)  0.070 | 1.05  (0.76 – 1.45)  0.764 | 0.007  (-0.040 — 0.055)  0.766 |
|  | 1 steps | | 1.42  (1.06 – 1.92)  0.021 | 0.044  (0.002 — 0.086)  0.038 | 1.04  (0.74 – 1.46)  0.826 | 0.007  (-0.050 — 0.064)  0.819 |
|  | 2 steps | | 1.45  (1.08 – 1.95)  0.015 | 0.049  (0.004 — 0.095)  0.035 | 0.86  (0.74 – 0.99)  0.039 | -0.024  (-0.050 — 0.001)  0.060 |
|  | 3 steps | | 1.18  (0.85 – 1.63)  0.325 | 0.019  (-0.024 — 0.063)  0.386 | 0.95  (0.71 – 1.27)  0.716 | -0.011  (-0.065 — 0.044)  0.702 |
|  | 4 steps | | 0.92  (0.60 – 1.41)  0.708 | -0.013  (-0.070 — 0.044)  0.657 | 0.88  (0.70 – 1.09)  0.240 | -0.022  (-0.058 — 0.013)  0.210 |
|  | 5 steps | | 0.97  (0.67 – 1.39)  0.852 | -0.005  (-0.047 — 0.036)  0.798 | 1.11  (0.91 – 1.36)  0.294 | 0.032  (-0.030 — 0.094)  0.308 |
|  | 6 steps | | 0.41  (0.24 – 0.69)  0.001 | -0.069  (-0.131 — -0.006)  0.031 | 0.63  (0.53 – 0.76)  <0.001 | -0.114  (-0.176 — -0.053)  <0.001 |
| Time step | | |  |  |  |  |
|  | Step 0 | | 1 (base) | 1 (base) | 1 (base) | 1 (base) |
|  | Step 1 | | 1.41  (1.07 – 1.85)  0.014 | 0.031  (0.009 — 0.052)  0.005 | 0.76  (0.53 – 1.07)  0.115 | -0.030  (-0.068 — 0.008)  0.121 |
|  | Step 2 | | 1.33  (0.75 – 2.35)  0.328 | 0.025  (-0.027 — 0.076)  0.350 | 1.09  (0.74 – 1.60)  0.659 | 0.010  (-0.040 — 0.060)  0.684 |
|  | Step 3 | | 1.36  (0.93 – 1.99)  0.109 | 0.027  (-0.003 — 0.058)  0.073 | 1.25  (0.85 – 1.86)  0.257 | 0.031  (-0.024 — 0.086)  0.266 |
|  | Step 4 | | 1.45  (0.91 – 2.30)  0.114 | 0.034  (-0.004 — 0.073)  0.081 | 1.35  (0.88 – 2.07)  0.168 | 0.043  (-0.016 — 0.102)  0.150 |
|  | Step 5 | | 1.76  (1.17 – 2.66)  0.007 | 0.062  (0.022 — 0.101)  0.002 | 1.27  (1.11 – 1.48)  0.001 | 0.035  (0.016 — 0.054)  <0.001 |
|  | Step 6 | | 1.67  (1.09 – 2.56)  0.019 | 0.053  (0.011 — 0.094)  0.012 | 1.46  (1.05 – 2.03)  0.025 | 0.053  (0.004 — 0.102)  0.033 |
|  | Step 7 | | 1.50  (0.81 – 2.75)  0.195 | 0.038  (-0.021 — 0.097)  0.210 | 2.66  (1.99 – 3.56)  <0.001 | 0.197  (0.124 — 0.269)  <0.001 |
| Random effects (95% CI) | | |  |  |  |  |
|  | | Clinic | 0.0127  (0.0012 – 0.1348) | 0.0002  (0.0000 — 0.0030) | 1.74*e^-33^  (1.50*e^-33^ – 2.02*e^-33^) | 5.43*e^-34^  (9.78*e^-47^ — 3.01*e^-21^) |
|  | | Clinic-time |  |  |  |  |
|  | | Individual |  |  |  |  |
| Log pseudolikelihood | | | -2276.62 | -1704.65 | -2420.40 | -2169.21 |
| Akaike information criteria | | | 4565.24 | 3421.30 | 4852.79 | 4350.43 |

Model 1 is the basic Hussey & Hughes model with fixed effects for time step, adjustment for clustering with random effects for clinic, and cluster robust standard errors.

CI, 95% confidence interval; CQI, Continuous Quality Improvement; RD, risk difference; RR, relative risk.

We describe the methods for these additional analyses in the Methods section (**lines 367-370**):

“Analysis of effect heterogeneity: We measured CQI effect heterogeneity by duration of CQI exposure, using the same mixed effects regression models as in the primary analyses but replacing the fixed effect for overall CQI exposure with fixed effects for CQI exposure for each time step since rollover to CQI.

And the additional results in the Results section (**lines 483-495**):

“CQI effect heterogeneity: There was variability in CQI effect on VL monitoring with time since rollover to the intervention, in all three mixed effects models. Immediately after rolling over to the intervention, the relative effect size was 1.17 (*p*=0.071) in Model 1. After two months of CQI exposure (lag of one step) and four months (lag of two steps), the relative effect sizes were 1.42 (*p*=0.021) and 1.45 (*p*=0.015) respectively. There was no significant CQI effect beyond six months of exposure (**Table 5**). Similarly, the absolute effect sizes varied with the same durations of exposure to CQI in Model 1: 1.7 percentage points (*p*=0.070) with immediate exposure, 4.4 percentage points (*p*=0.038) after two months of exposure, 4.9 percentage points (*p*=0.035) after four months of exposure and no significant change beyond six months of exposure (**Table 5**). The results were essentially the same with Models 2 and 3 for relative and absolute effect sizes. There was no heterogeneity in CQI effect on repeat HIV testing by duration of CQI exposure in either relative or absolute measures (**Table 5**).

And Discussion (**lines 524-533**):

“We also found heterogeneous effects of CQI on VL monitoring by duration of the cluster’s exposure to CQI: effects reached their maximum two to four months after intervention rollover and waned thereafter. The time pattern we see here can be explained by a ‘natural course’ of innovation — initially health workers need to learn to implement the innovation to maximum effectiveness; over time, the need for the innovation declines (e.g. novel processes supporting VL monitoring) as more and more patients have benefited. At the same time, the waning effects following a maximum could also indicate health worker fatigue in implementing an innovation that has now become routine. This latter explanation would argue for implementation research on approaches to maintain long-term innovation effects. ”

*2.14. Modelling visits and time. In their analysis section (page 13) the authors state that they used a fixed effect for time step. I do not see how this can be used when the women had in principle many visits spanning the study period and most of them had four or more. This is also tied to the issue of whether to include a random effect for woman. Clinic level clustering accounts, as far as I can see, for the fact that women within cluster are more similar than women between clusters. It does not account for whether women who were more likely to have a VL monitoring at time i were also more likely to have one at time j.*

**Author response:** We have now fitted two additional regression models with different random effects to evaluate the robustness of our main findings. We are very reassured that our findings are indeed robust and reproducible. The three models are presented in **Table 3**.

We have added these details to the manuscript as follows:

Methods (**lines 346-356**):

“We used three models to estimate the effect of the CQI intervention. First, we used the standard Hussey and Hughes model, which includes clinic random effects and time step fixed effects (Model 1, **Table 3**) [31]. Second, we used an extension to this model with a random clinic-time step interaction term (Model 2, **Table 3**). This extension is recommended by Hemming et al [32], because it allows secular time trends to vary randomly by clinic. Third, we extended the standard Hussey and Hughes model with individual random effects nested within clinic random effects (Model 3, **Table 3**). This model accounts not only for clustering of outcomes by clinic but also for clustering of outcomes within individual women across time steps. In all models, we used cluster robust standard errors to further adjust for clustering and any model misspecification [19, 20].”

And Results (**lines 440-495**):

“*Effect of CQI on HIV care tests*

Primary analyses - pregnant women living with HIV: Women exposed to CQI were significantly more likely to receive a VL test: the relative effect sizes (relative risks, RRs) in our three primary analyses were essentially equal, ranging from 1.38 to 1.39 (**Table 3**, **Figure 4**), and were all highly significant (all *p*<0.001). Similarly, the absolute effect sizes of receiving a VL test per ANC visit were very similar in all three primary analyses, 3.9 to 4.1 percentage points (**Table 4**) in the intervention compared to the control arm. With a median of six ANC visits per pregnancy in this study, the absolute effect size per visit translates to a cumulative probability of receiving a VL test in pregnancy of 58% in the intervention arm compared to 46% in the control arm. With the recent WHO recommendations for at least eight ANC visits [6], these values are equivalent to a cumulative probability of receiving a VL test of 69% in the intervention arm compared to 57% in the control arm.

Primary analyses - pregnant women not living with HIV: CQI did not have a statistically significant effect on repeat HIV testing in any of our three primary analyses. The RRs ranged from 1.00 to 1.01, and the corresponding *p*-values ranged from 0.877 to 0.958 (**Table 3**, **Figure 4**). These numbers are equivalent to an absolute change in the probability of receiving a repeat HIV test per ANC visit of 0.0 to 0.3 percentage points (all *p*≥0.758, **Table 4**). With a median of six ANC visits in this study, this effect size per visit translates to a 59-60% cumulative probability of receiving a repeat HIV test during pregnancy in both intervention and control arms. With the WHO-recommended eight ANC visits, these numbers translate to 69-70% cumulative probability in both arms.

Sensitivity analyses: When we adjusted for additional covariates, all effect size estimates remained essentially the same as in the primary analyses. RRs for HIV VL monitoring ranged from 1.36 to 1.37 (all *p*<0.001) with adjustments for maternal age and parity; 1.39 to 1.40 (*p*-values <0.001 to 0.001) with adjustments for gestation at each visit and total number of ANC visits; and 1.35 to 1.36 (*p*-values 0.005 to 0.009) with adjustments for all four covariates. The absolute effect sizes for VL monitoring per ANC visit were 3.7 to 4.0 percentage points higher (all *p*<0.001) with adjustments for maternal age and parity; 4.4 to 4.6 percentage points (all *p*<0.001) with adjustments for gestation at each visit and total number of ANC visits; and 4.2 to 4.4 percentage points (all *p*≤0.008) with adjustments for all four covariates.

RRs for repeat HIV testing ranged from 1.00 to 1.01 (all *p* ≥0.871) with adjustments for maternal age and parity; 1.08 (all *p*≥0.525) with adjustments for gestation at each visit and total number of ANC visits; and 1.08 (all *p*≥0.504) with adjustments for all four covariates. The absolute effect sizes for repeat HIV testing per ANC visit ranged from 0.0 to 0.3 percentage points (all *p*≥0.757) with adjustments for maternal age and parity; 1.1 to 1.3 percentage points (all *p*≥0.457) with adjustments for gestation at each visit and total number of ANC visits; and 1.1 to 1.3 percentage points (all *p*≥ 0.445) with adjustments for all four covariates.

CQI effect heterogeneity: There was variability in CQI effect on VL monitoring with time since rollover to the intervention, in all three mixed effects models. Immediately after rolling over to the intervention, the relative effect size was 1.17 (*p*=0.071) in Model 1. After two months of CQI exposure (lag of one step) and four months (lag of two steps), the relative effect sizes were 1.42 (*p*=0.021) and 1.45 (*p*=0.015) respectively. There was no significant CQI effect beyond six months of exposure (**Table 5**). Similarly, the absolute effect sizes varied with the same durations of exposure to CQI in Model 1: 1.7 percentage points (*p*=0.070) with immediate exposure, 4.4 percentage points (*p*=0.038) after two months of exposure, 4.9 percentage points (*p*=0.035) after four months of exposure and no significant change beyond six months of exposure (**Table 5**). The results were essentially the same with Models 2 and 3 for relative and absolute effect sizes. There was no heterogeneity in CQI effect on repeat HIV testing by duration of CQI exposure in either relative or absolute measures (**Table 5**).”

**Reviewer 3**

*3.0. This manuscript presents the results of a stepped-wedge cluster randomised trial in South Africa. The trial compared a CQI intervention to standard of care in nurse-led antenatal care facilities with the aim of improving viral load testing among women living with HIV, and repeat HIV testing among women who were HIV negative at presentation for antenatal care.*

*This is a clearly thought out, pragmatic study which is well described in the paper. The*

*findings show that CQI can improve the completion of viral load testing during pregnancy in*

*low-resource and high-burden settings. The lack of effect of repeat HIV testing is clearly*

*considered and raises important issues around the broader context within which CQI*

*interventions are implemented.*

*Overall, this is a well written manuscript that offers important insights into both the potential*

*for CQI interventions in primary care settings and the challenges with implementing and*

*evaluating CQI interventions. I have a few minor comments and points of clarification.*

**Author response:** We thank Reviewer 3 for his/her encouraging feedback.

*3.1. Page 7 line 115. Study setting: here you say 6 clinics but the abstract and description said 7 clinics (6 clusters – 2 small clinics combined).*

**Author response:** We have now clarified our sentence regarding the number of clinics as follows (**lines 166-170**):

“Our CQI intervention was conducted at seven nurse-led National Department of Health (DoH) primary healthcare clinics: six were located within the geographic bounds of the AHRI Population Intervention Platform Surveillance Area (PIPSA) South [39], and one clinic was located in the market town of Mtubatuba, which is often used by PIPSA residents (**Figure 1**).”

*3.2. Page 11. Data sources: If I understand correctly, both completion of maternal viral loads and repeat HIV testing were ascertained from the maternity case record retrospectively at the time of delivery. My experience is that the MCRs are very well completed but are there any concerns about data quality in these patient held records? What happened if the MCR was lost/ had to be replaced and there was missing data for a period of the pregnancy? It might be useful to add something about the quality of the outcome data to the methods/ discussion.*

**Author response:** Yes, all HIV care tests were ascertained based on MCR documentation. Although the MCRs were informative, we were concerned about data quality: as indicated in Table 2, only ~half of VL tests performed had a documented result, raising concerns for incomplete documentation of other aspects of clinical care.

We have now added further comments to the Results and Discussion (Strengths and limitations section):

Results (**lines** **398-405**):

“Most maternity case records contained clinical information. Among the 2498 women recruited for this study (**Figure 3**), five had blank maternity case records because their previous records had been lost, and a further 19 had blank records because they never attended ANC. Of all women recorded in our study database, 2160 participants were eligible for our primary ITT analysis contributing 13,212 observations during pregnancy; 338 women were excluded from our primary analysis because their HIV status was unknown, their first antenatal clinic was not a study clinic, or their first antenatal clinic was unknown (**Figure 3**).”

Discussion (**lines 679-685**):

“Second, we measured our endpoints as documented in the maternity case records. Although most maternity case records contained clinical information, the poor documentation of VL results among women who had a VL performed raises concerns for data quality overall. It is thus possible that actual repeat HIV testing and VL monitoring rates were higher than measured in our study albeit not documented. In this case, we would also expect that the effect sizes that we have measured are underestimates of the true effect sizes.”

*3.3. Page 14 line 300/301. Unsure what is meant by HIV diagnosis coverage of HIV-positive women?*

**Author response:** We have now improved our description of HIV diagnosis coverage which relates to the proportion of women living with HIV aware of their status (based on HIV status documented in the maternity case record) as follows:

We have now reframed the sentence as follows (**lines 406-409**):

“We estimated the total number of women living with HIV who attended a study clinic at their first ANC visit to be 1026 (1011 with documented HIV status + 15 women with unknown HIV status by delivery), of whom 99% were diagnosed HIV-positive – and therefore had opportunity to start ART.”

*3.4. Line 306. Usually avoid starting a sentence with a number (50.3%..)*

**Author response:** We have now systematically checked the remainder of the paper and corrected this. The sentence now reads (**line 416**):

*“*Around half the women (50.3%, 95% CI 45.1-55.4%) attended their first ANC visit before 20 weeks’ gestation.*”*

*3.5. Line 341. Missing a bracket.*

**Author response:** This has now been corrected. We have also carefully checked all instances of brackets throughout the paper to make sure that no opening or closing brackets are missing.

*3.6. Discussion. Line 364. I agree with the authors discussion of the providers perceived importance of repeat HIV testing compared to viral load. I wonder if there is also any differences from the patient side. Are there any issues with women refusing HIV testing or perhaps not being aware that it should happen so not asking if it isn't done? In contrast*

*perhaps women living with HIV are more aware of the impact of having a viral load test.*

**Author response:** This is an important consideration. There are indeed plausible factors from the patients’ side which may also explain the differences in CQI effect on our endpoints.

We have now added these points to our Discussion as follows (**lines 541-556**):

“CQI activities aimed at increasing repeat HIV testing may thus not have resonated as strongly with health workers as did CQI activities aimed at improving VL monitoring. A related issue is patient perceptions or behaviours. For example, women not living with HIV may have less perceived risk of infection [40], or concerns about negative partner attitudes if they received a positive HIV diagnosis [41]. Presenting late to ANC for the first time may have also been a barrier for some women not living with HIV to receive a second HIV test given the guidelines for testing every three months. Women not living with HIV may have left the clinic prior to receiving an HIV test due to long queues at these busy clinics, as identified in our process evaluation [12]. In another submitted manuscript reviewing women’s knowledge of HIV care at six weeks postpartum, we found that most women — those living with and those not living with HIV — were knowledgeable about testing for HIV (regardless of health status), the role of ART and CD4 counts; yet even among women living with HIV, knowledge of the role of VL monitoring was very low [42]. Thus, it is unlikely that patient knowledge (lacking or present) would have influenced our endpoints despite our expectations to the contrary.”

*3.7. Lines 371-374. Not sure I clearly understand what the authors mean here. Do you mean the CQI intervention was practically easier, with the barriers to viral load testing easier to identify and address than the barriers to repeat HIV testing. Perhaps the CQI intervention "favoured" the viral load testing outcome in this way in so far as it was the lower hanging fruit of things to address in the clinics?*

**Author response:** We thank Reviewer 3 for raising this point. We believe that VL monitoring involved more complex clinical processes and more staff cadres who may have functioned as a ‘safety net’ to identify eligible women for testing. On the other hand, repeat HIV testing had fewer processes and staff involved, with fewer areas for improvement.

We have now clarified this point further (**lines 560-576**):

“….. it might have been practically easier to identify and address problems in achieving high levels of coverage with VL monitoring compared with repeat HIV testing because VL monitoring involves more processes and more complexity than repeat HIV testing – thus, CQI which addresses process quality, had fewer opportunities to change practice in repeat HIV testing. For instance, VL monitoring involved identifying women eligible for the test, phlebotomy, following up and documenting results, and retrospectively capturing routine ART clinical data onto the national monitoring and evaluation database (TIER.Net) – most of which could be intervened on through CQI. During each visit, women living with HIV may have interacted with different staff cadres in the clinic: nurses for clinical management, lay counsellors for adherence counselling – with additional opportunities to identify eligible women for testing during the clinic appointment. Other staff (e.g. data capturers) may have also identified women eligible for VL monitoring while filing previous VL results or capturing routine data onto TIER.Net after the visit. Conversely, repeat HIV testing involved simpler workflow: identifying eligible women, conducting the rapid test and immediately documenting the result, with only one type of staff cadre involved (usually lay counsellor).”

*3.8. Lines 375-378. This is a really important issue. Staff turnover and buy-in pose a major threat to CQI interventions and this requires considering for the roll out of these interventions in low-resource health systems.*

**Author response:** We thank Reviewer 3 for this feedback. We further emphasized this point as follows:

Discussion (**lines 701-706**):

“It is also unclear as to how far clinic health workers ‘normalise’ CQI into their practice once the CQI mentors have completed an engagement, particularly given the human resource shortages and staff turnover we are aware of. Knowledge of time to assimilation of CQI could inform future study designs which account for a suitable time lag before outcomes can be expected to change.”

*3.9. Lines 394-405. The authors discuss here the issue of selecting a more proximal outcome measure. Do the authors have any suggestions here for future research based on your experiences? What sorts of outcomes might be important to consider when evaluating and monitoring the success of CQI interventions?*

**Author response:** We have now added the following to the discussion (**lines 715-725**):

“Outcome measures that lie on plausible causal pathways from an exposure to an outcome can be used to empirically confirm mechanistic hypotheses. CQI is an intervention that can work its way to outcomes through multiple pathways, because it empowers local health workers to identify and implement those health systems improvements and innovations that are most promising in the particular contexts in which they work. In this case – and other complex interventions –, local health workers can identify likely mechanisms connecting an intervention to outcomes. In the case of HIV VL monitoring and repeat HIV testing, proximal endpoints lying on the causal pathways from CQI could include availability of test kits, presence of systems for patient tracking and tracing, health worker motivation to test, [12]and patient demand for testing.

*3.10. Lines 429-430. Is there a reason you do not account for a delay in intervention effect/time for the intervention to stabilise, perhaps in sensitivity analyses?*

**Author response:** Thank you very much for the excellent suggestion. We have now added a sub-analysis to measure CQI effect heterogeneity by time since rollover to the intervention (in time steps). This analysis is presented in the following sections:

Methods (**lines 367-372**):

“Analysis of effect heterogeneity: We measured CQI effect heterogeneity by duration of CQI exposure, using the same mixed effects regression models as in the primary analyses but replacing the fixed effect for overall CQI exposure with fixed effects for CQI exposure for each time step since rollover to CQI.

We used Stata version 15.0 (StataCorp. 2017. College Station, TX: StataCorp LLC) for all statistical analyses.”

Results (**lines 483-495**):

“CQI effect heterogeneity: There was variability in CQI effect on VL monitoring with time since rollover to the intervention, in all three mixed effects models. Immediately after rolling over to the intervention, the relative effect size was 1.17 (*p*=0.071) in Model 1. After two months of CQI exposure (lag of one step) and four months (lag of two steps), the relative effect sizes were 1.42 (*p*=0.021) and 1.45 (*p*=0.015) respectively. There was no significant CQI effect beyond six months of exposure (**Table 5**). Similarly, the absolute effect sizes varied with the same durations of exposure to CQI in Model 1: 1.7 percentage points (*p*=0.070) with immediate exposure, 4.4 percentage points (*p*=0.038) after two months of exposure, 4.9 percentage points (*p*=0.035) after four months of exposure and no significant change beyond six months of exposure (**Table 5**). The results were essentially the same with Models 2 and 3 for relative and absolute effect sizes. There was no heterogeneity in CQI effect on repeat HIV testing by duration of CQI exposure in either relative or absolute measures (**Table 5**)

And Discussion (**lines 524-533**):

We also found heterogeneous effects of CQI on VL monitoring by duration of the cluster’s exposure to CQI: effects reached their maximum two to four months after intervention rollover and waned thereafter. The time pattern we see here can be explained by a ‘natural course’ of innovation — initially health workers need to learn to implement the innovation to maximum effectiveness; over time, the need for the innovation declines (e.g. novel processes supporting VL monitoring) as more and more patients have benefited. At the same time, the waning effects following a maximum could also indicate health worker fatigue in implementing an innovation that has now become routine. This latter explanation would argue for implementation research on approaches to maintain long-term innovation effects.”

*3.11. Lastly, the inclusion of the TIDieR framework is really helpful to think about how this or similar interventions could be scaled. The success of the intervention is dependent on the skills of the CQI mentors who deliver the intervention and how they engage with the health providers. Some discussion on issues around identifying/training suitable CQI mentors when scaling/reproducing this sort of intervention might be useful.*

**Author response:** We thank Reviewer 3 for raising this point pertinent to success and sustainability of such a complex intervention in other resource-poor settings.

We have now added the following to the Discussion (**lines 625-658**):

“The key resource needed to implement CQI is CQI mentors’ and clinic health workers’ time. CQI mentors typically spent several hours per week training and supporting clinic health workers during the intensive phase of CQI; during the maintenance phase, the mentors spent substantially less time interacting with the clinic health workers, typically several hours per month. The training sessions at the very beginning of each step and the action learning sessions each required an entire day. CQI activities required time to (i) identify drivers of poor VL monitoring and repeat HIV testing, (ii) conduct group meetings to design clinic-specific solutions, (iii) implement those solutions (e.g. different methods to track women eligible for HIV care tests), and (iv) use clinic-based real-time data sources to test whether their solutions worked towards target testing rates.

CQI mentors who are adaptable and relate professionally and culturally to health workers at target health facilities, may be more successful at engaging health workers to implement CQI. Mentors must be adept at generating descriptive data, and ideally familiar with implementing the desired change in practice. Obtaining basic CQI certification may take around 18 hours (online)[13] to three days [14] of coursework.

In addition to the CQI mentors’ time and the health workers’ time, a so-called Improvement Advisor – a more highly qualified health professional than the CQI mentors with the ability to coach and lead the CQI mentors – is an integral part of the team to oversee success. In our experience, the Improvement Advisor would need to invest around 20% of their time; in other settings this would depend on the needs of the CQI mentors and participating clinics. Thus, the time – and financial – costs of the intervention were overall low. Feasibility and scalability of CQI will, however, not only depend on resource requirements but also on clinic health workers’ motivation and mindset. CQI requires changes in individual behaviour and institutional practice, which in turn are driven by job satisfaction, ambition, experience and local workplace culture and constraints [50].

In our study, the CQI mentors typically held CQI meetings 2-3 times per week during the intensive two-month step. For routine implementation at larger scale, alternative meeting frequencies may also work. For instance, it may be more practicable to conduct visits less frequently (e.g. once per week) but over a longer period of time (e.g. half a year). The optimal frequency of mentor visits will likely depend on how many clinic health workers are available for CQI activities and whether the clinic activities that the CQI chose for improvement require additional support.”

**References**

1.	Mate KS, Ngubane G, Barker PM. A quality improvement model for the rapid scale-up of a program to prevent mother-to-child HIV transmission in South Africa. International Journal for Quality in Health Care. 2013;25(4):373-80. doi: 10.1093/intqhc/mzt039. PubMed PMID: 23710069;

2.	Singh K, Brodish P, Speizer I, Barker P, Amenga-Etego I, Dasoberi I, et al. Can a quality improvement project impact maternal and child health outcomes at scale in northern Ghana? Health Research Policy and Systems. 2016;14(1):45. doi: 10.1186/s12961-016-0115-2. PubMed PMID: 27306769;

3.	Magge H, Kiflie A, Mulissa Z, Abate M, Biadgo A, Bitewulign B, et al. Launching the Ethiopia Health Care Quality Initiative: Interim Results and Initial Lessons Learned. BMJ Open Quality. 2017;6(Suppl 1):A1-A39.

4.	Mutanda P, Muange P, Lutta M, Kinyua K, Chebet L, Okaka B, et al. Improving prevention of mother to child transmission of HIV care: Experiences from implementing quality improvement in Kenya. NASCOP, PEPFAR, USAID, IHI, 2017.

5.	Bhardwaj S, Barron P, Pillay Y, Treger-Slavin L, Robinson P, Goga A, et al. Elimination of mother-to-child transmission of HIV in South Africa: Rapid scale-up using quality improvement. South African Medical Journal. 2014;104(3):239-43. doi: 10.7196/samj.7605. PubMed PMID: 24893500;

6.	WHO. WHO recommendations on antenatal care for a positive pregnancy experience. Geneva: World Health Organization, 2016.

7.	Colbourn T, Nambiar B, Bondo A, Makwenda C, Tsetekani E, Makonda-Ridley A, et al. Effects of quality improvement in health facilities and community mobilization through women's groups on maternal, neonatal and perinatal mortality in three districts of Malawi: MaiKhanda, a cluster randomized controlled effectiveness trial. International Health. 2013;5(3):180-95. Epub 2013/09/14. doi: 10.1093/inthealth/iht011. PubMed PMID: 24030269;

8.	Oyeledun B, Phillips A, Oronsaye F, Alo OD, Shaffer N, Osibo B, et al. The Effect of a Continuous Quality Improvement Intervention on Retention-In-Care at 6 Months Postpartum in a PMTCT Program in Northern Nigeria: Results of a Cluster Randomized Controlled Study. Journal of Acquired Immune Deficiency Syndromes. 2017;75(Suppl 2):S156-S64. doi: 10.1097/QAI.0000000000001363. PubMed PMID: 28498185;

9.	Cawley C, McRobie E, Oti S, Njamwea B, Nyaguara A, Odhiambo F, et al. Identifying gaps in HIV policy and practice along the HIV care continuum: evidence from a national policy review and health facility surveys in urban and rural Kenya. Health Policy and Planning. 2017;32(9):1316-26. Epub 2017/10/06. doi: 10.1093/heapol/czx091. PubMed PMID: 28981667;

10.	Ezeanolue EE, Powell BJ, Patel D, Olutola A, Obiefune M, Dakum P, et al. Identifying and Prioritizing Implementation Barriers, Gaps, and Strategies Through the Nigeria Implementation Science Alliance: Getting to Zero in the Prevention of Mother-to-Child Transmission of HIV. Journal of Acquired Immune Deficiency Syndromes. 2016;72 Suppl 2:S161-6. doi: 10.1097/QAI.0000000000001066. PubMed PMID: 27355504;

11.	Proctor E, Silmere H, Raghavan R, Hovmand P, Aarons G, Bunger A, et al. Outcomes for implementation research: conceptual distinctions, measurement challenges, and research agenda. Administration and Policy in Mental Health and Mental Health Services. 2011;38(2):65-76. Epub 2010/10/20. doi: 10.1007/s10488-010-0319-7. PubMed PMID: 20957426;

12.	Yapa HM, Dhlomo-Mphatswe W, Moshabela M, De Neve J-W, Herbst C, Jiamsakul A, et al. A Continuous Quality Improvement intervention to improve antenatal HIV care testing in rural South Africa: evaluation of implementation in a real-world setting. [submitted]. 2019.

13.	Institute for Healthcare Improvement I. IHI Open School Online Courses 2020. Available from: <http://www.ihi.org/education/IHIOpenSchool/Courses/Pages/OpenSchoolCertificates.aspx>.

14.	Institute for Healthcare Improvement I. In-Person Training: Breakthrough Series College 2020. Available from: <http://www.ihi.org/education/InPersonTraining/breakthrough-series-college/Pages/default.aspx>.

15.	Michel J, Chimbindi N, Mohlakoana N, Orgill M, Bärnighausen T, Obrist B, et al. How and why policy-practice gaps come about: a South African universal health coverage context. Journal of Global Health Reports. 2020;3:e2019069. doi: 10.29392/joghr.3.e2019069.

16.	Grol R, Wensing M. Implementation of Change in Healthcare: a Complex Problem. In: Grol R, Wensing M, Eccles M, Davis D, editors. Improving Patient Care: The Implementation of Change in Health Care. 2nd ed. West Sussex, UK: BMJ Books, Wiley Blackwell; 2013.

17.	Chetty T, Yapa HMN, Herbst C, Geldsetzer P, Naidu KK, De Neve J-W, et al. The MONARCH intervention to enhance the quality of antenatal and postnatal primary health services in rural South Africa: protocol for a stepped-wedge cluster-randomised controlled trial. BMC health services research. 2018;(18):625. doi: 10.1186/s12913-018-3404-3. PubMed PMID: 30089485;

18.	Hemming K, Taljaard M, McKenzie JE, Hooper R, Copas A, Thompson JA, et al. Reporting of stepped wedge cluster randomised trials: extension of the CONSORT 2010 statement with explanation and elaboration. British Medical Journal. 2018;363:k1614. doi: 10.1136/bmj.k1614. PubMed PMID: 30413417;

19.	Zou G. A Modified Poisson Regression Approach to Prospective Studies with Binary Data. American Journal of Epidemiology. 2004;159(7):702-6. doi: 10.1093/aje/kwh090. PubMed PMID: 15033648;

20.	Zou GY, Donner A. Extension of the modified Poisson regression model to prospective studies with correlated binary data. Statistical Methods in Medical Research. 2011;22(6):661-70. doi: 10.1177/0962280211427759. PubMed PMID: 22072596;

21.	Cummings P. The Relative Merits of Risk Ratios and Odds Ratios. Archives of Pediatric and Adolescent Medicine. 2009;163(5):438-45. doi: 10.1001/archpediatrics.2009.31. PubMed PMID: 19414690;

22.	Greenland S. Interpretation and choice of effect measures in epidemiologic analyses. American Journal of Epidemiology. 1987;125(5):761-8. Epub 1987/05/01. doi: 10.1093/oxfordjournals.aje.a114593. PubMed PMID: 3551588;

23.	Hauck WW, Anderson S, Marcus SM. Should We Adjust for Covariates in Nonlinear Regression Analyses of Randomized Trials? Controlled Clinical Trials. 1998;19:249-56. doi: 10.1016/s0197-2456(97)00147-5. PubMed PMID: 9620808;

24.	Hernan MA, Clayton D, Keiding N. The Simpson's paradox unraveled. International Journal of Epidemiology. 2011;40(3):780-5. Epub 2011/04/02. doi: 10.1093/ije/dyr041. PubMed PMID: 21454324;

25.	Yelland L, Salter A, Philip R. Relative risk restimation in cluster randomized trials: a comparison of generalized estimating equation methods. International Journal of Biostatistics. 2011;7(1):1-26.

26.	Carter RE, Lipsitz SR, Tilley BC. Quasi-likelihood estimation for relative risk regression models. Biostatistics. 2005;6(1):39-44. Epub 2004/12/25. doi: 10.1093/biostatistics/kxh016. PubMed PMID: 15618526;

27.	Williamson T, Eliasziw M, Fick GH. Log-binomial models: exploring failed convergence. Emerging Themes in Epidemiology. 2013;10(1):14. Epub 2013/12/18. doi: 10.1186/1742-7622-10-14. PubMed PMID: 24330636;

28.	Chen W, Qian L, Shi J, Franklin M. Comparing performance between log-binomial and robust Poisson regression models for estimating risk ratios under model misspecification. BMC Medical Research Methodology. 2018;18(1):63. Epub 2018/06/23. doi: 10.1186/s12874-018-0519-5. PubMed PMID: 29929477;

29.	Zou GY. Assessment of risks by predicting counterfactuals. Statistics in Medicine. 2009;28(30):3761-81. Epub 2009/10/27. doi: 10.1002/sim.3751. PubMed PMID: 19856279;

30.	Marschner IC, Gillett AC. Relative risk regression: reliable and flexible methods for log-binomial models. Biostatistics. 2012;13(1):179-92. Epub 2011/09/15. doi: 10.1093/biostatistics/kxr030. PubMed PMID: 21914729;

31.	Hussey MA, Hughes JP. Design and analysis of stepped wedge cluster randomized trials. Contemporary Clinical Trials. 2007;28(2):182-91. doi: 10.1016/j.cct.2006.05.007. PubMed PMID: 16829207;

32.	Hemming K, Taljaard M, Forbes A. Analysis of cluster randomised stepped wedge trials with repeated cross-sectional samples. Trials. 2017;18(1):101. doi: 10.1186/s13063-017-1833-7. PubMed PMID: 28259174;

33.	Greenland S, Rothman KJ. Measures of Occurrence. In: Rothman KJ, Greenland S, editors. Modern Epidemiology. Philadelphia: Lippincott Williams and Wilkins; 2008. p. 33-51.

34.	Hernan MA, Clayton D, Keiding N. The Simpson's paradox unraveled. International Journal of Epidemiology. 2011;40(3):780-5. Epub 2011/04/02. doi: 10.1093/ije/dyr041. PubMed PMID: 21454324;

35.	Yelland LN, Salter AB, Ryan P. Performance of the modified Poisson regression approach for estimating relative risks from clustered prospective data. American Journal of Epidemiology. 2011;174(8):984-92. Epub 2011/08/16. doi: 10.1093/aje/kwr183. PubMed PMID: 21841157;

36.	Ukoumunne OC, Forbes AB, Carlin JB, Gulliford MC. Comparison of the risk difference, risk ratio and odds ratio scales for quantifying the unadjusted intervention effect in cluster randomized trials. Statistics in Medicine. 2008;27(25):5143-55. Epub 2008/07/10. doi: 10.1002/sim.3359. PubMed PMID: 18613226;

37.	MSF. Getting to undetectable: usage of HIV viral load monitoring in five countries. Geneva: Médecins Sans Frontières, 2014.

38.	Awungafac G, Amin ET, Fualefac A, Takah NF, Agyingi LA, Nwobegahay J, et al. Viral load testing and the use of test results for clinical decision making for HIV treatment in Cameroon: An insight into the clinic-laboratory interface. PloS one. 2018;13(6). doi: 10.1371/journal.pone.0198686. PubMed PMID: 29889862;

39.	Tanser F, Hosegood V, Barnighausen T, Herbst K, Nyirenda M, Muhwava W, et al. Cohort Profile: Africa Centre Demographic Information System (ACDIS) and population-based HIV survey. International Journal of Epidemiology. 2008;37(5):956-62. Epub 2007/11/14. doi: 10.1093/ije/dym211. PubMed PMID: 17998242;

40.	Alemu YM, Ambaw F, Wilder-Smith A. Utilization of HIV testing services among pregnant mothers in low income primary care settings in northern Ethiopia: a cross sectional study. BMC Pregnancy and Childbirth. 2017;17(1):199. Epub 2017/06/26. doi: 10.1186/s12884-017-1389-2. PubMed PMID: 28646888;

41.	Gebremedhin KB, Tian B, Tang C, Zhang X, Yisma E, Wang H. Factors associated with acceptance of provider-initiated HIV testing and counseling among pregnant women in Ethiopia. Patient Preference and Adherence. 2018;12:183-91. Epub 2018/02/09. doi: 10.2147/PPA.S148687. PubMed PMID: 29416320;

42.	Yapa HM, Drayne R, Klein N, De Neve J-W, Petoumenos K, Jiamsakul A, et al. Infant feeding knowledge and practice vary by maternal HIV status: a nested cohort study in rural South Africa. [submitted]. 2020.
