# Supplementary material for: The impact of continuous quality improvement on coverage of antenatal HIV care tests in rural South Africa: Results of a stepped-wedge cluster-randomised controlled implementation trial
Source: PLoS Med. 2020 Oct 7;17(10):e1003150. doi: 10.1371/journal.pmed.1003150 (PMC7540892; doi:10.1371/journal.pmed.1003150)
Supplement: Attachment — Submitted filename: Author response to reviewers_MONARCH_Yapa et al_14Jun2020.docx [file pmed.1003150.s008.docx]

14 June 2020

Dear Richard,

**Re: PMEDICINE-D-19-03348**

**Title:** “The impact of Continuous Quality Improvement on coverage of antenatal HIV care tests in rural South Africa: results of a stepped-wedge cluster-randomised controlled implementation trial”

We thank the editors and reviewers for their comments. In response, we have further improved our manuscript.

In particular, we have added a TIDieR table to the main manuscript to describe details of the intervention, and added further text to the intervention description section in the manuscript.

Below, we describe in detail how we have responded to each comment. We show the comments in italics and our responses in normal font. We show the changes that we have made indented and in quotation marks, with line numbers that refer to the revised version of our manuscript.

Sincerely,

H. Manisha Yapa, Jan-Walter de Neve, Terusha Chetty, Carina Herbst, Frank Post, Awachana Jiamsakul, Pascal Geldsetzer, Guy Harling, Wendy Dhlomo-Mphatswe, Mosa Moshabela, Philippa Matthews, Osondu Ogbuoji, Frank Tanser, Dickman Gareta, Kobus Herbst, Deenan Pillay, Sally Wyke, Till Bärnighausen

**EDITOR COMMENTS**

*1. In your data statement, please briefly explain the reasons why data cannot be made available (e.g. restrictions imposed by the study ethics approval).*

The study data can indeed be made available to scientists requesting access. The data policy at the Wellcome Trust’s Africa Health Research Institute (AHRI) in South Africa, where this study took place, prescribes that access to data from any study conducted at AHRI must be requested through a simple centralized institutional process. Data requests to AHRI are only very rarely denied.

We have improved our explanation of data access as follows:

“Access to the study data is governed by the data access policy at the AHRI, the institution where this study took place. Access will be granted upon request. Such requests can be made to the AHRI Data Repository via the AHRI website <https://www.ahri.org/research/>. Researchers who meet the criteria for access to fully anonymised patient level data will be granted data access."

*2. Please quote the number of participants in each study arm in your abstract; and add a few words to note that no adverse events were reported.*

We have now added this information to the abstract (**lines 30-32**):

“Between 15 July 2015 and 30 January 2017, 2160 participants with 13,212 ANC visits (intervention *n*=6877, control *n*=6335) were eligible for ITT analysis. No adverse events were reported.”

*3. Please trim the author summary, ensuring that the three subsections contain 3-4 points, each of 1-2 short sentences.*

We have now trimmed the author summary to a maximum of 4 bullet points per subsection as recommended:

“**Why was this study done?**

- Gaps in implementation of evidence-based guidelines can slow progress towards major health systems goals, such as the elimination of mother-to-child transmission of HIV.
- Continuous Quality Improvement (CQI) has the potential to improve service delivery within available resources, and has been successful in resource-rich settings.
- There is very limited evidence on the impact of CQI in improving health systems in resource-poor primary care.

**What did the researchers do and find?**

- We assigned seven public-sector primary care clinics in rural South Africa to receive CQI in random sequence.
- The intervention was delivered by a trained team of local CQI mentors who worked collaboratively with clinic health workers. They aimed to address root causes of poor HIV viral load (VL) monitoring among pregnant women living with HIV and repeat HIV testing among pregnant women not living with HIV.
- CQI increased HIV VL monitoring by nearly 40%, but did not improve repeat HIV testing.
- Lay counsellors, who conducted HIV testing and counselling in the clinics, were redeployed during the study period, placing a strain on available human resources for HIV testing.

**What do these findings mean?**

- We found that CQI can be effective in improving quality of primary care in resource-poor communities.
- It may have been easier to improve HIV VL monitoring than repeat HIV testing, because the baseline attainment of VL monitoring was far lower.
- Resource shortages, particularly staffing shortages, may have prevented CQI from achieving its full potential.
- Future research should focus on the conditions under which CQI is most likely to be successful.”

4*. Please remove the sentence of discussion at line 152.*

We have now removed the sentence at line 152 as recommended. The last paragraph of the Introduction now reads **(lines 145-149)**:

“Our stepped-wedge cluster RCT aimed to establish the effectiveness of CQI on important proximate indicators of the quality of HIV-related ANC in primary care. We hypothesized that a CQI intervention would be effective at improving (i) VL monitoring in pregnant women living with HIV and (ii) repeat HIV testing in pregnant women not living with HIV.”

*5. In your reference list, please make sure that journal names are abbreviated consistently (including “PLoS Med.”, for example).*

We have carefully check all references and all journal names are now consistently abbreviated.

*6. Please clarify the situation with regard to reference 65.*

Reference 65 has undergone peer review at another journal and we have been invited to revise and resubmit the manuscript. Given that *PLoS Medicine* cannot include manuscripts not yet accepted for publication in the reference list, we have now removed the relevant text from the Discussion.

The text we have now removed is (lines 646-652):

“In another submitted manuscript reviewing women’s knowledge of HIV care at six weeks postpartum, we found that most women — those living with and those not living with HIV — were knowledgeable about testing for HIV (regardless of health status), the role of ART and CD4 counts; yet even among women living with HIV, knowledge of the role of VL monitoring was very low [65]. Thus, it is unlikely that patient knowledge (lacking or present) would have influenced our endpoints despite our expectations to the contrary.”

We have accordingly also removed reference 40 (which we were also invited to revise and resubmit) and adjusted the text in the manuscript as follows:

**(Lines 293-301):**

“We also conducted a process evaluation to better understand intervention delivery and explain our primary findings. For this we sourced field notes and reports by the CRH team collated every two months. The reports described actual visit dates and type, results of the root-cause analyses, the improvement interventions (including PDSA cycles), successes and challenges, as well as other observations including impressions of health worker receptivity to CQI (**Table 1**). We further conducted semi-structured interviews with consenting health workers on their experiences of implementing CQI. We describe in detail the methods, data, and results of the process evaluation in an upcoming scientific publication.”

**(Lines 234-429):**

“Finally, clinic health workers urged the CRH team to continue to work with them on quality improvement beyond the end of this study. We will report further details on these findings in an upcoming publication on the process evaluation that accompanied this trial. Preliminary results of *Situational Analyses* conducted prior to intervention rollover, identified staffing shortages and lack of a formal tracking system for patients eligible for HIV care tests or their results at most clinics (**Table 1**).”

**(Lines 708-714):**

“Clinic health workers could not always participate in CQI meetings and failed to complete some of the CQI tasks, despite their general enthusiasm for the intervention. We will report further details on these results in an upcoming publication on the process evaluation accompanying this trial. The reasons for these deviations were mostly rooted in structural capacity constraints in this rural South African health system, including staffing shortages, rapid staff turnover, and competing clinical commitments [68].”

**(Lines 717-720):**

“The solutions that each clinic identified and implemented did indeed differ, even though all participating clinics were located in the same district in rural KwaZulu-Natal. We will report detailed data in an upcoming publication on the process evaluation that accompanied this trial.”

*7. Please rename the attachments with completed checklists (e.g. “S1_CONSORT_Checklist”).*

We have renamed our two supplementary attachments as follows:

- “S1_Text”
- S1_Table
- S1_Fig.tif
- S2_Table
- S3_CONSORT_checklist”

**REVIEWER COMMENTS**

**Reviewer 1**

*The remarks made at first reading have all been taken into account and the answers are well argued. I have no additional comments.*

**Author response:** We thank Reviewer 1 for the positive feedback.

**Reviewer 2**

*The authors have addressed all my points.*

**Author response:** We thank Reviewer 2 for the positive feedback.

**Reviewer 3**

*The authors have thoroughly and thoughtfully responded to my comments and those raised by the other reviewers. I have no additional comments.*

**Author response:** We thank Reviewer 3 for the positive feedback.

**Reviewer 4**

*This reviewer did not review the original paper but two related things in my mind attenuate the potential impact of this manuscript. First, the CQI strategy is very sparsely described. The literature on QI across a large number of disease conditions (e.g. stroke, HIV) as well as a variety of settings and organizations is remarkably heterogeneous in clinical effects. To advance the science of quality improvement strategies, research on such approaches must be crystal clear about the nature of the quality improvement intervention in order to make its effects (or absence thereof) interpretable. I did not see where in the manuscript enough detail on who the CRH team was composed of (credentials? training?), when were they supposed to go to facilities (what was the dose?) and how much leeway they had; what the specific actions taken were (presumably training, mentoring, teaching etc). Second, the implementation outcomes are likewise underdeveloped particularly from a quantitative perspective. What fraction of intended mentoring or coaching visits were made? Who received the team? What evidence of implementation outcomes or process outcomes were present? Also, although not all of this is in the scope of perhaps this manuscript — implementation outcomes at any or all of these levels would not only be useful and interesting but indeed critical for making this study a contribution to the literature. Finally context is critical and although there are organizational scales that are difficult to use, even survey results like how leadership at the facility level perceived in QI activities could be useful. In addition to Proctors “specifying” implementation strategies guidance, this paper should also make use of standards for reporting quality improvement studies* [*http://www.squire-statement.org/*](http://www.squire-statement.org/) *. So in short, while I applaud the rigorous design, the impact on the field in general and on the CQI literature would be enhanced with these considerations in my view. I would encourage addition of these data to results if possible, and if not, discussion and consideration for a future submission on these outcomes alone.*

**Author response:** We thank Reviewer 4 for his/her constructive and insightful comments. We have added a Template for Intervention Description and Replication (TIDieR) table to the main manuscript, which describes in detail all components of this CQI intervention.

**Table 1. MONARCH CQI intervention description: Template for Intervention Description and Replication (TIDieR) framework**

| **Name (1)** | Management and Optimisation of Nutrition, Antenatal Care, Reproductive, Child Health and HIV services (MONARCH) implementation project |
| --- | --- |
| **Why (2)** | EMTCT requires rigorous implementation of national treatment and care guidelines whilst respecting and protecting the human rights of women living with HIV. Yet in resource-poor settings gaps in guidelines implementation may hinder progress towards eMTCT. |
|  | CQI has the potential to improve clinical processes within a given set of resources and has been shown to support guidelines implementation in resource-rich settings, but there is a paucity of rigorous evidence in resource-poor settings. |
|  | The CQI intervention was designed to improve implementation of South African eMTCT guidelines in rural primary health care clinics, with a focus on improved diagnosis of incident HIV infection (through repeat HIV testing in pregnancy) and better monitoring of HIV viral load in pregnancy. |
| **What, materials (3)** | CQI as a complex behavioural intervention draws on several impact theories, particularly Total Quality Management (TQM), Normalisation Process Theory (NPT) and educational and motivational theories [40]. CQI aims to improve quality of organizational processes with a patient-centred focus, utilising objective information (real-time data). |
|  | CQI uses a structured ‘form’: a set of **standard tools** which are used flexibly by facilitators and thought to function differently in different contexts. The tools include: |
|  | - Process Maps [36]: which aim to understand existing clinic systems by documenting clinic workflow on specific activities (e.g. VL monitoring). These serve to target improvement activities |
|  | - Fishbone Diagrams [37]: which allow analysis of root causes of a target outcome, by pre-specified categories (e.g. patient-level, clinic-level, health worker-level). |
|  | - Plan-Do-Study-Act (PDSA) cycles [38]: which are iterative action cycles starting with a specific aim and well-defined process change activity. The process change is adopted, adapted or abandoned based on findings of the review phase (Study) of the cycle. |
|  | - Run Charts [39]: which are plotted during improvement activities using clinic-level data, monitor target outcome time trends (e.g. VL monitoring), and provide visual feedback on trend changes due to the intervention. |
|  | **Hard copies of the following documents were also used:** |
|  | - South African eMTCT guidelines at each clinic (provided by the South African National Department of Health, DoH) |
|  | - DoH eMTCT clinical care monitoring forms |
|  | - DoH monthly tally sheets (for monthly collation of DoH monitoring and evaluation (M&E) indicators) |
|  | - DoH routine M&E clinic registers: to identify ANC patients eligible for VL monitoring and repeat HIV testing |
|  | **Other items required:** |
|  | - A projector for presentations |
|  | - Stationery for using CQI tools (above) and note taking |
|  | - Refreshments for CQI introductory and action learning meetings to encourage and thank staff |
| **What, procedures (4); when and how much (8)** | The CQI intervention targeted health workers at clinics and delivered in two phases: intensive and maintenance. |
|  | **Intensive** |
|  | *Initial induction visits:* |
|  | - During two weeks leading up to scheduled intervention step rollover date |
|  | - Up to five visits in each clinic lasting 1-4 hours except for the very first visit (whole day) |
|  | - Introduce the team and initiate relationships, introduce CQI, undertake *Situational Analysis* (understand resources and existing clinical processes), baseline data analysis and review quality of clinic data sources (e.g. registers) |
|  | *Intervention visits:* |
|  | - During the two-month intervention step |
|  | - Up to seven intervention visits: each was expected to last 1-4 hours. Mentors used CQI tools flexibly to identify clinic-specific gaps in implementing eMTCT guidelines and brainstorm solutions. |
|  | *Support visits:* |
|  | - During the two-month intervention step: each was expected to last 1-2 hours. |
|  | - Up to 12 support visits in each clinic during the Intensive phase |
|  | - Provided ongoing support on progress and review of PDSA cycles |
|  | - *Action learning sessions:* |
|  | - Held at the end of each two-month intervention step: each was expected to last a whole day |
|  | - The aim was to consolidate learning and facilitate buy-in of the next randomised clinic |
|  |  |
|  | **Maintenance** |
|  | *Support visits or Maintenance visits:* |
|  | - Monthly after the two-month intervention step: each was expected to last 1-2 hours. |
|  | - Varied total number of visits depending on intervention rollover date - up to 12 for first randomised clinic |
|  | - Provide ongoing support on progress, review of PDSA cycles, ongoing mentorship to resolve problems and support to improve other relevant aspects of clinical care (e.g. infant HIV PCR testing) if time permitted |
| **Who provided (5)** | The CQI intervention was delivered in person by the University of KwaZulu-Natal (UKZN) Centre for Rural Health (CRH) CQI mentors who travelled from Durban, KwaZulu-Natal, to the study site. They had previous experience in a large CQI project on prevention of MTCT elsewhere in South Africa. |
|  | The CRH mentors consisted of two isiZulu-speaking South African nurses and a data capturer who carried out field activities, with close support from an Improvement Advisor (a consultant obstetrician with CQI experience), Scientific Advisor and data manager. |
|  | Apart from study objectives and study design, no additional training was provided to the CRH team. Their competence in delivering the intervention was not formally assessed. |
| **How (6)** | The CRH mentors identified a team of clinic-based health workers willing to participate in CQI activities (clinic CQI team). Health workers working in areas relevant to the intervention were selected by the clinic Operational Manager. The CRH mentors aimed to recruit health workers able to commit to group CQI activities for most of the study period to facilitate sustainability. |
| **Where (7)** | Clinic visits were conducted in a meeting room at each clinic. |
|  | Action learning sessions were held at larger venues including the AHRI auditorium given the larger number of participants. |
| **Tailoring (9)** | Process changes to be implemented varied by clinic depending on findings of root-cause analyses. All standard CQI tools were implemented at all clinics. |
|  | Time constraints imposed by the study design resulted in having to prioritise interventions: where the Situational Analysis identified multiple systemic gaps, some factors such as M&E data quality could not be addressed as rigorously as others. Tailoring was guided by the CRH Improvement Advisor. |
| **Modification (10)** | No modifications were made. |
| **How well, planned (11)** | A schedule of visits was planned with a standard ‘dose’ for each intervention step, prior to the first scheduled clinic rollover. |
|  | *We assessed intervention adherence* by comparing the planned schedule of visits (and type) against CRH mentor reports submitted at the end of each intervention step. The reports contained information on each clinic as follows: |
|  | - Actual visit dates, visit type (Items (4) & (8) above), and clinic CQI team attendees |
|  | - Process maps |
|  | - Situational analysis findings including barriers to VL monitoring and repeat HIV testing |
|  | - PDSA cycles implemented and outcomes |
|  | - Successes and challenges implementing CQI |
| **How well, actual (12)** | *‘Dose’:* The overall ‘dose’ was greater than planned at the outset. Actual visit summaries compared with scheduled visits will be reported in a separate process evaluation. |
|  | *‘Reach’:* All seven facilities were enthusiastic about CQI. |
|  | *Fidelity*: Staffing shortages were an important feature noted during Situational Analyses across all clinics – this may have affected their availability to consistently attend CQI meetings and CQI implementation. DoH redeployment of lay counsellors may have affected HIV testing practices. Almost all clinics had no formal patient tracking system to identify women eligible women for HIV care tests prior to CQI intervention rollover. |

AHRI, Africa Health Research Institute; ANC, antenatal care; EMTCT, elimination of mother-to-child transmission of HIV; CQI, Continuous Quality Improvement; DoH, South African National Department of Health; HIV PCR, polymerase chain reaction (nucleic acid amplification test for detecting HIV infection); M&E, monitoring & evaluation; MTCT, mother-to-child transmission of HIV; VL, HIV viral load.

We have further added the following paragraphs to the Methods section to provide additional information on the intervention context, which is not featured in the TIDieR table (**Lines 168-175):**

“Additional contextual information such as laboratory results workflow, staffing (including lay counsellors) and clinic size, is described in **Supplementary File 1**. The intervention was delivered by an external CQI team of mentors (including two isiZulu speaking nurses) from the Centre for Rural Health (CRH) at the University of KwaZulu-Natal, who travelled to the study site (hereafter referred to as the CRH team). The mentors were closely supported by an Improvement Advisor (consultant obstetrician), a Scientific Advisor, and a data manager.“

**(Lines 243-252):**

*“Delivering the intervention according to the stepped-wedge study design* is detailed in **Table 1**. CQI was delivered intensively to each cluster during the two-month intervention step and followed by a less intensive maintenance phase (**Table 1, Figure 2**). A standard ‘dose’ of ~19 visits was planned for the Intervention phase (2-3 visits per week) and ~monthly visits in the Maintenance phase (**Figure 2**) for ongoing support and mentorship. Action learning sessions were held at the end of each Intervention step, only for randomised clinics given the study design. **Table 1** further describes materials, procedures, how and where the intervention was delivered (including duration and timing of CQI visits), and how we measured dose, reach and fidelity of the intervention. We describe details of implementation including fraction of actual mentoring visits (‘dose’), health worker attendance at mentoring visits (‘reach’) and fidelity of the intervention in a linked process evaluation [40].”

**(Lines 293-301):**

“We also conducted a process evaluation to better understand intervention delivery and explain our primary findings. For this we sourced field notes and reports by the CRH team collated every two months. The reports described actual visit dates and type, results of the root-cause analyses, the improvement interventions (including PDSA cycles), successes and challenges, as well as other observations including impressions of health worker receptivity to CQI (**Table 1**). We further conducted semi-structured interviews with consenting health workers on their experiences of implementing CQI. We describe in detail the methods, data, and results of the process evaluation in an upcoming scientific publication. “

We have also improved our discussion of several implementation issues in reference to our process evaluation in the Discussion as follows:

**(Lines 696-699):**

“We observed that health workers maintained positive attitudes towards CQI and our CQI mentors during the study. In particular, health workers were enthusiastic to learn CQI tools and improve quality of services, and were keen to continue working with the CRH team.”

**(Lines 705-720):**

“Based on our observations, CQI mentors implemented the CQI intervention with high fidelity. However, clinic health workers’ implementation of the CQI process and techniques was of somewhat lower fidelity. Clinic health workers could not always participate in CQI meetings and failed to complete some of the CQI tasks, despite their general enthusiasm for the intervention. We will report further details on these results in an upcoming publication on the process evaluation accompanying this trial. The reasons for these deviations were mostly rooted in structural capacity constraints in this rural South African health system, including staffing shortages, rapid staff turnover, and competing clinical commitments [68].

The CQI process and management techniques are intended to be the same across clinics and communities; the solutions that they identify, test and revise are intended to be tailored to each clinic and will thus differ across contexts. The solutions that each clinic identified and implemented did indeed differ, even though all participating clinics were located in the same district in rural KwaZulu-Natal. We will report detailed data in an upcoming publication on the process evaluation that accompanied this trial.”

**(Lines 737-747):**

“In addition to the CQI mentors’ time and the health workers’ time, a so-called Improvement Advisor – a more highly qualified health professional than the CQI mentors with the ability to coach and lead the CQI mentors – is an integral part of the team to oversee success. In our experience, the Improvement Advisor would need to invest around 20% of their time; in other settings this would depend on the needs of the CQI mentors and participating clinics. Thus, the time – and financial – costs of the intervention were overall low. Feasibility and scalability of CQI will, however, not only depend on resource requirements but also on clinic health workers’ motivation and mindset. CQI requires changes in individual behaviours and institutional practices, which in turn are determined by job satisfaction, ambition, experience and local workplace culture and constraints [50].”
